# Advances in Synthesis of π-Extended Benzosilole Derivatives and Their Analogs

**DOI:** 10.3390/molecules25030548

**Published:** 2020-01-27

**Authors:** Tuan Thanh Dang, Hue Minh Thi Nguyen, Hien Nguyen, Tran Ngoc Dung, Minh Tho Nguyen, Wim Dehaen

**Affiliations:** 1 Department of Chemistry, Hanoi University of Science, Vietnam National University (VNU), 19 Le Thanh Tong, Ha Noi 100000, Vietnam; dangthanhtuan@gmail.com; 2 Department of Chemistry and Center for Computational Science, Hanoi National University of Education, 136 Xuan Thuy, Ha Noi 100000, Vietnam; hiennguyendhsphn@gmail.com (H.N.); trandung171@gmail.com (T.N.D.); 3 Institute for Computational Science and Technology (ICST), Ho Chi Minh City 700000, Vietnam; 4 Department of Chemistry, KU Leuven, Celestijnenlaan 200F, B-3001 Leuven, Belgium; minh.nguyen@kuleuven.be

**Keywords:** benzosiloles, fused benzosiloles, dibenzosilole, 9-silafluorenes, optoelectronic materials, silylation reactions

## Abstract

Benzosiloles and their π-extended derivatives are present in many important advanced materials due to their excellent physical properties. Especially, they have found many potential applications in the development of novel electronic materials such as OLEDs, semiconductors and solar cells. In this review, we have summarized several main approaches to construct (di)benzosilole derivatives and (benzo)siloles fused to aromatic five- and six-membered heterocycles.

## 1. Introduction

Benzosiloles and their π-extended derivatives have attracted much attention because of their extraordinary properties in comparison with other well-known organic materials [1,2,3,4,5,6,7,8,9,10,11,12,13,14,15,16,17]. There are two types of π-extended benzosilole derivatives classified as ladder-type and spiro-type structures. Especially, dibenzosiloles (9-silafluorenes) possess unique photophysical and electronic properties such as high electron-transporting performances which could also be used in promising photovoltaic organic materials [1,2,3,4,5,6,7,8,9,10,11,12,13,14,15,16,17]. In fact, they are important motifs appearing as key building blocks in the structure of many kinds of novel light emitting materials, solar cells, semiconductors, electroluminescent materials [1,2,3,4,5,6,7,8,9,10,11,12,13,14,15,16]. Therefore, a lot of new synthetic methods for the formation of dibenzosilole derivatives have been widely developed [18,19,20]. Conventional methods for the synthesis of dibenzosilole derivatives are often based on the metal-halide exchange of dihalobiarenes, followed by cyclization with dichlorosilanes [21,22,23,24]. These reactions are often carried out under very low temperature conditions and/or for a long reaction time. On the other hand, this method may be disadvantageous in the synthesis of (di)benzosilole derivatives containing sensitive functional groups, which is often limiting further applications. In the light of recent advances in the field of transition metals catalysis, many new structures and applications of dibenzosilole analogs have been demonstrated, which contributed to the development of materials science [18,19]. In addition, the new cyclization reactions via a silyl radical process have given more efficient and sustainable approaches to benzosilole and dibenzosilole derivatives under mild conditions [20].

## 2. Synthesis of π-Extended Benzosiloles

### 2.1. Conventional Metal-Halide Exchange Method

**Scheme 1:**
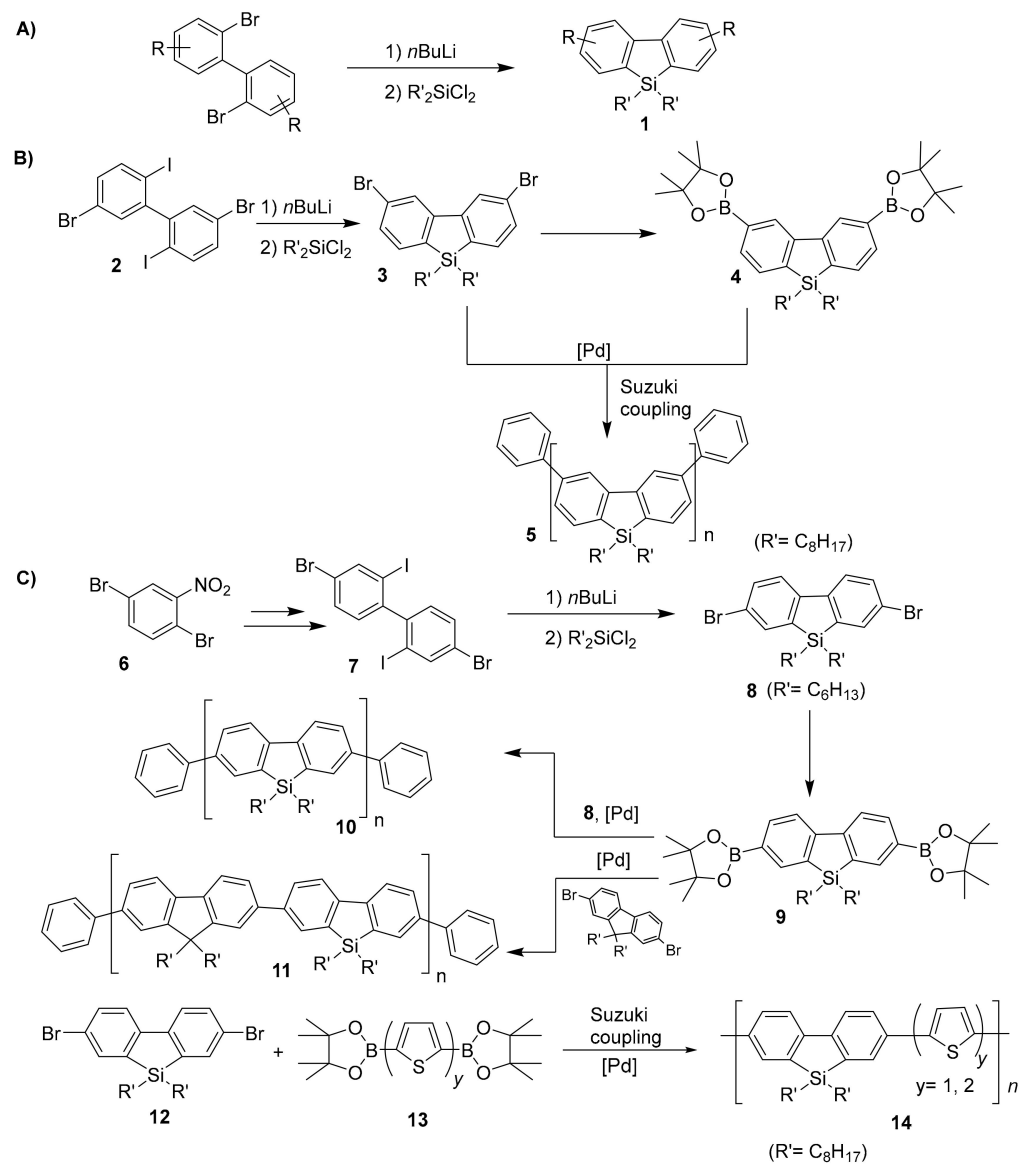
Synthesis of dibenzosilole derivatives and dibenzosilole-containing polymers.

In 1955, the first synthesis of 9,9-diphenylsilafluorene was reported by Gilman and Gorsich [25]. In this seminal publication, 2,2′-dilithiumdiphenyl was prepared by the lithium-bromide exchange of the corresponding dibromobiphenyl **1** with *n*BuLi, and then reacted with one equivalent of diphenyldichlorosilane to afford to 9,9-diphenysilafluorene (**2**) in 49% yield (Scheme 1, pathway A). Afterwards, there have been several modified procedures giving better yields of 9-silafluorene derivatives [21,22,23,24]. Due to excellent optoelectronic properties of 9-silafluorenes, the incorporation of 9-silafluorenes and related compounds in polymers have currently become an important topic in materials science [5]. Using this metal-halide exchange strategy, a series of monomers containing dibrominated 9-silafluorenes were successfully prepared [21,22,23,24,25]. In 2005, Homes, Cao groups disclosed the synthesis of dibrominated 9-silafluorene monomers **4, 9** which were used in the polymerization (Scheme 1 pathways B, C) [26,27]. In fact, poly(3,6-dibenzosiloles) **5** were prepared via Suzuki coupling of **3** and **4** in the presence of Pd catalyst in very good yields. Mw and Mn of polymer **5** was reported to be 23000 and 11000, respectively. In 2006, Huang et al. reported a general strategy to access from **7** via biphenyl derivative **8** the 2,7-dibromo-9-silafluorenes **9** which can be used as key building blocks in polymer chemistry [28]. Thus, these building blocks were employed in the synthesis of poly(2,7-dibenzosiloles) materials which have been described to be a new class of high energy gap light emitting polymers (Scheme 1) [28]. In order to explore novel optoelectronic properties, the utilization of 9-silafluorene derivatives as key building blocks in the synthesis of copolymers also gained much attentions (Scheme 1, pathway C) [10]. As a result of the combination of several well-known monomers, a series of novel copolymers **11, 14** containing the 9-silafluorene moiety were successfully prepared using Pd-catalyzed cross-coupling reactions [11,14].

**Scheme 2:**
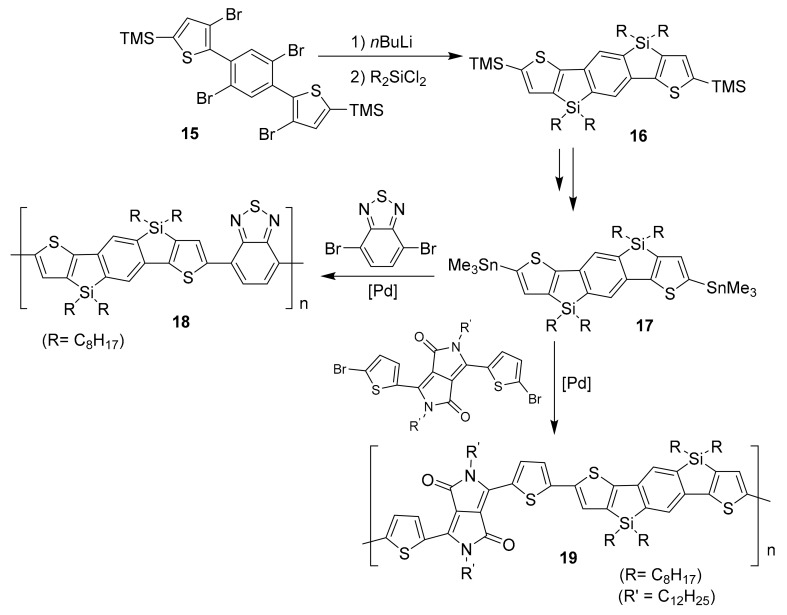
Synthesis of π-extended dibenzosiloles and polymers containing dibenzosilole unit.

Ashraf, Chen et al. reported an efficient synthesis of a novel fused coplanar chromophore, silaindacenodithiophene **16**, by the tetra-lithiation of 2,5-bis(3-bromo-2-thienyl)-1,4-dibromobenzene **15** and then a subsequent addition of dichloro di-*n*-octylsilane as shown in Scheme 2 [29]. This key building block was employed (after conversion to the bistin derivative **17**) in the synthesis of two silaindacenodithiophene (SiIDT) alternating copolymers **18** in field-effect transistors and photovoltaic devices. These polymers showed both very high ambipolar charge transport, with holes and electrons exhibiting mobilities and high solar cell efficiencies. At the same time, Jen and coworkers demonstrated the same approach in the synthesis of a silaindacenodithiophene monomer [30]. A similar Pd-catalysed synthesis of a new copolymer **19** which was used for the fabrication of solar cells by Stille coupling reaction has also been reported [31].

**Scheme 3:**
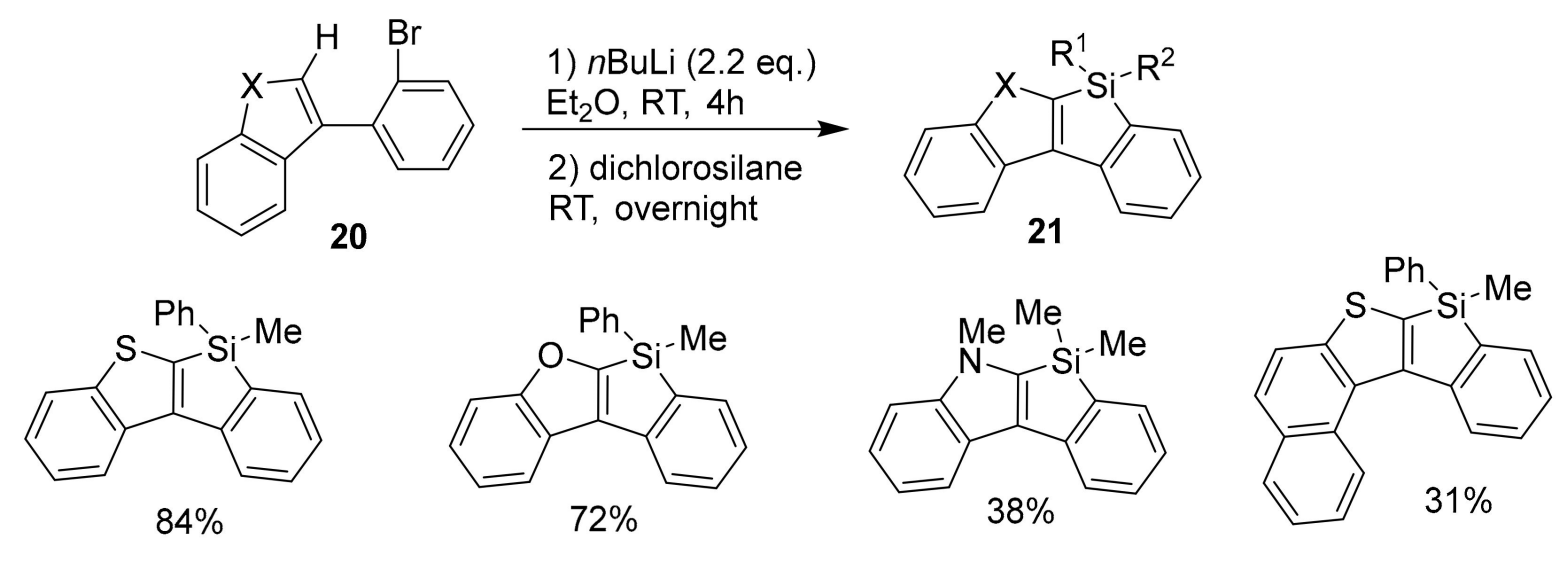
Synthesis of heterocycles-fused benzosiloles.

In 2017, Oestreich, Yamaguchi et al. reported a practical synthesis of heterocycle-fused benzosiloles **21** by two-fold metalation of benzo[b]thiophenes **20** followed by electrophilic substitutions with dichlorosilane derivatives (Scheme 3) [32]. The success of this procedure is based on the relatively high acidity of the C(sp^2^)–H bond at the C2 position of the benzothiophene ring and the metal-halide exchange at the C(sp^2^)–Br bond. A series of new benzothiophene-fused benzosiloles were successfully synthesized using this method.

**Scheme 4:**
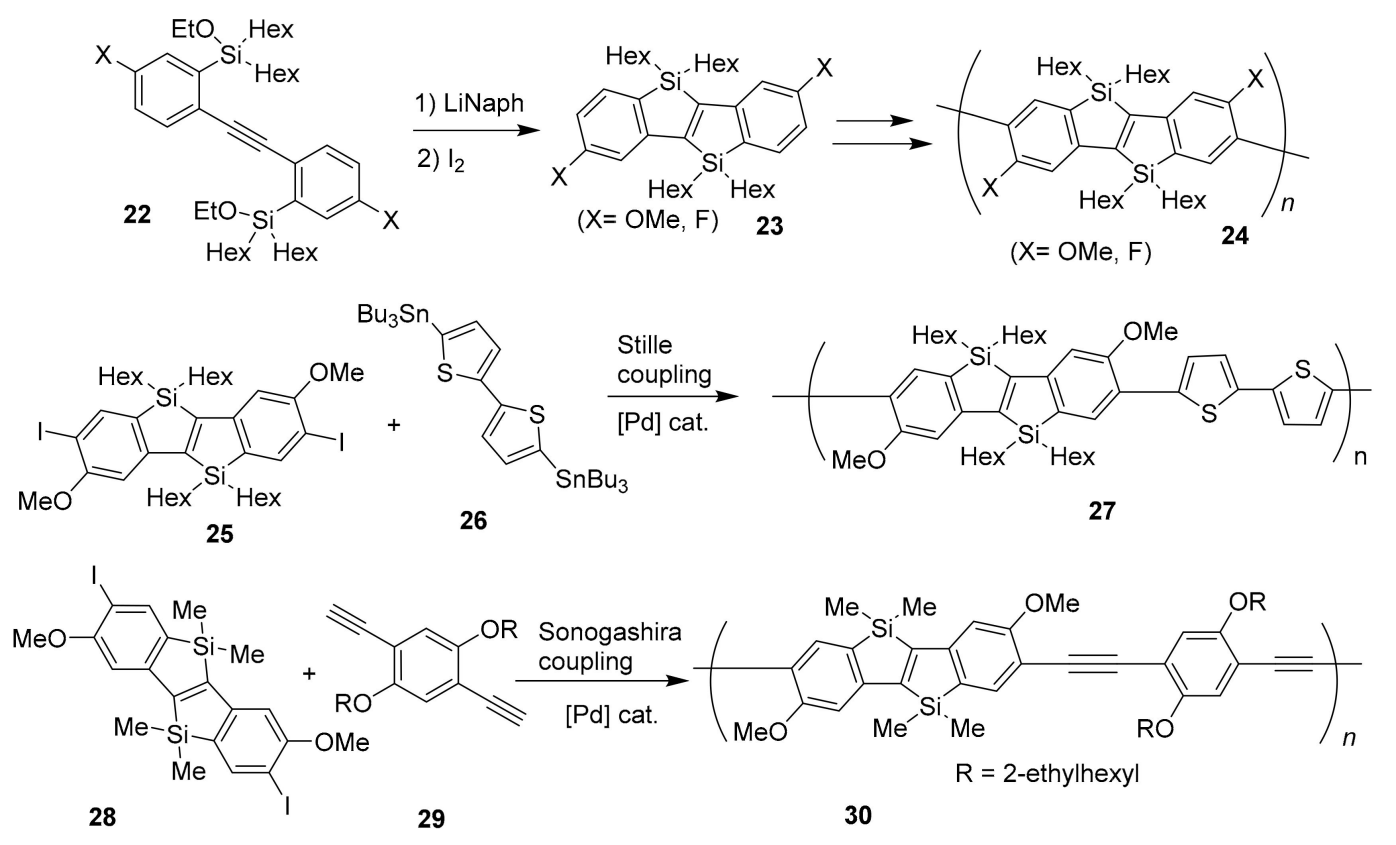
Synthesis of bis-silicon-bridged stilbenes and polymers containing bis-silicon-bridged stilbene moiety.

Especially, Yamaguchi et al. described a new synthetic method for the synthesis of *bis*-silicon-bridged stilbene **23** by intramolecular reductive cyclization of *bis*(*o*-silyphenyl)acetylene **22** using lithium naphthalenide (LiNaph) followed by the treatment with iodine (Scheme 4) [33]. After lithiations with *sec*-BuLi, 2,7-diiodinated derivatives **25** were prepared by the treatment with molecular iodine. Starting from 2,7-diiodinated derivative **25** and **28**, and building blocks **26** and **29**, a series of polymers/copolymers **24**, **27** and **30** were successfully synthesized by Pd-catalyzed cross-coupling polymerisations.

**Scheme 5:**
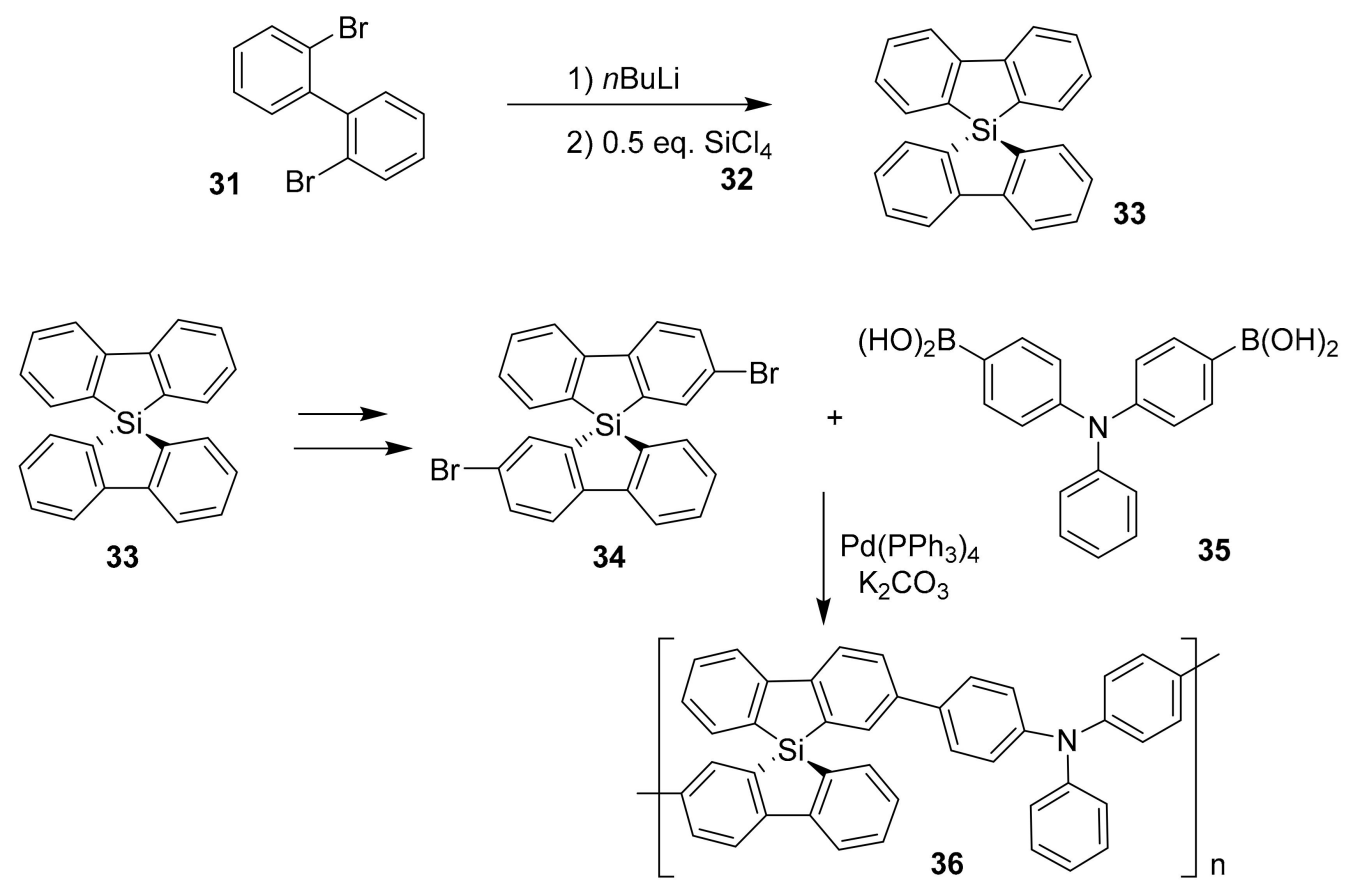
Synthesis of 9,9′-spiro-9-silabifluorenes.

In 1995, 9,9′-spiro-9-silabifluorene **33** was prepared from dibromobiphenyl **31** using a similar metal-halide exchange method. This molecule **33** showed high thermal stability (melting point is 227 °C) and could be used as the key building block in the structure of polymers, leading to an improvement in glass transition temperature (*T_g_*) and thermal stability (Scheme 5) [34]. Tian et al. developed a new copolymer **36** which combined 9,9′-spiro-9-silabifluorene and triphenylamine monomers via Suzuki cross coupling reaction of dibromo spirosilafluorene derivative **34** and triphenylamine diboronic acid **35** [35]. Several studies on molecular, thermal, optical and electroluminescent properties of **36** were carried out. The introduction of a spirosilafluorene as the key building block in this copolymer resulted in high thermal stability and good solubility in common organic solvents. This material showed a potential application as hole transport material in LED devices. Due to the extraordinary properties of 9,9′-spiro-9-silabifluorene, a series of novel derivatives of this molecule were synthesized by the introduction of different heteroatoms into the 9,9′-spiro-9-silabifluorene structure.

**Scheme 6:**
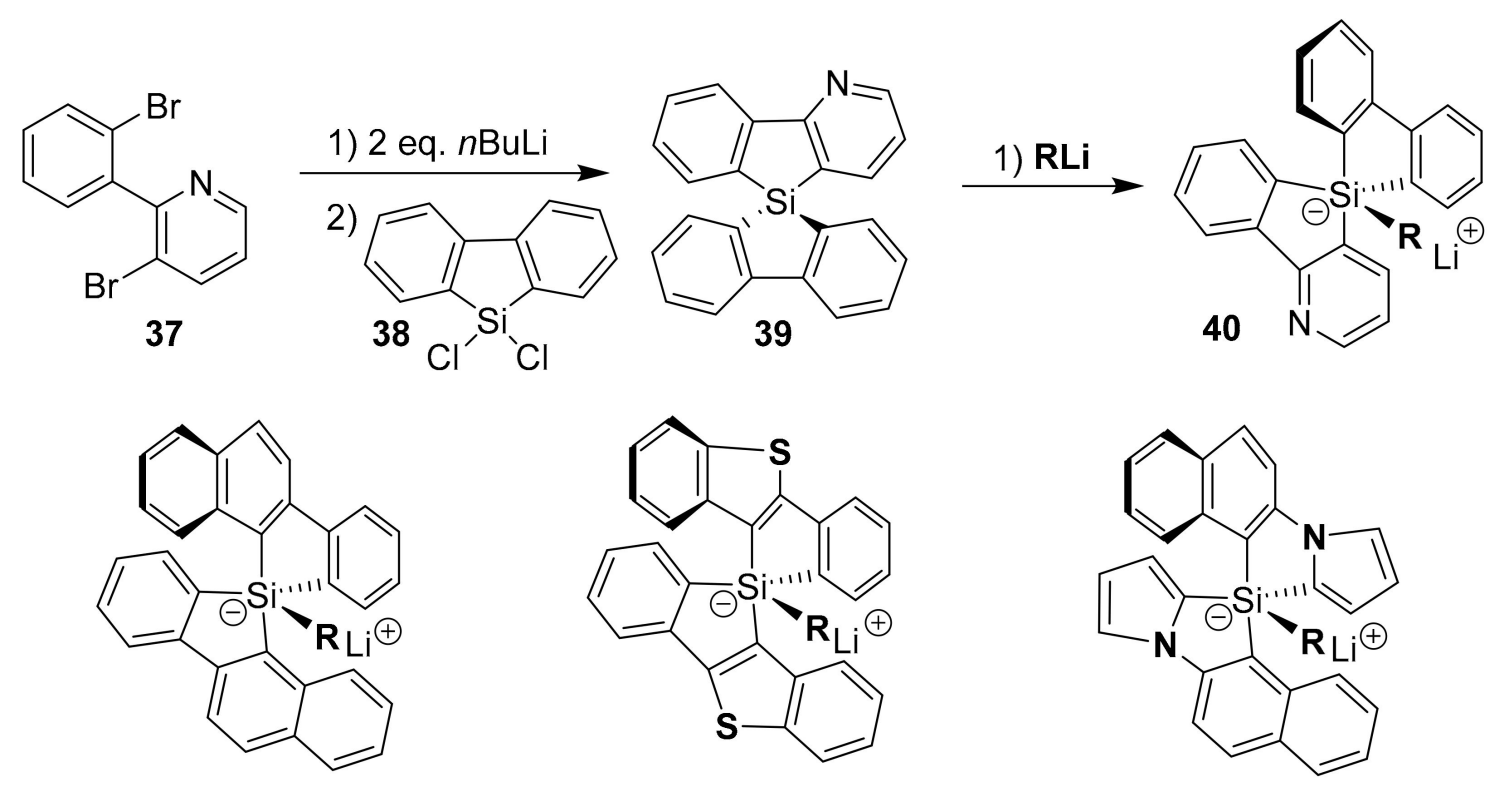
Synthesis of hypervalent silicon compounds.

Since the preparation of heterocycle-fused benzosiloles and 9,9′-spiro-9-silabifluorenes became routine and practical, the introduction of other heteroatoms into the structure of these molecules is easy to be realized [36] starting from the appropriate building blocks such as **37** and **38**. Interestingly, heterocycle-fused 9,9′-spiro-9-silabifluorenes **39** and other analogs can react with one more equivalent of organolithium compounds to form pentacoordinate systems (hypervalent silicon-containing compounds) (Scheme 6) [37].

**Scheme 7:**
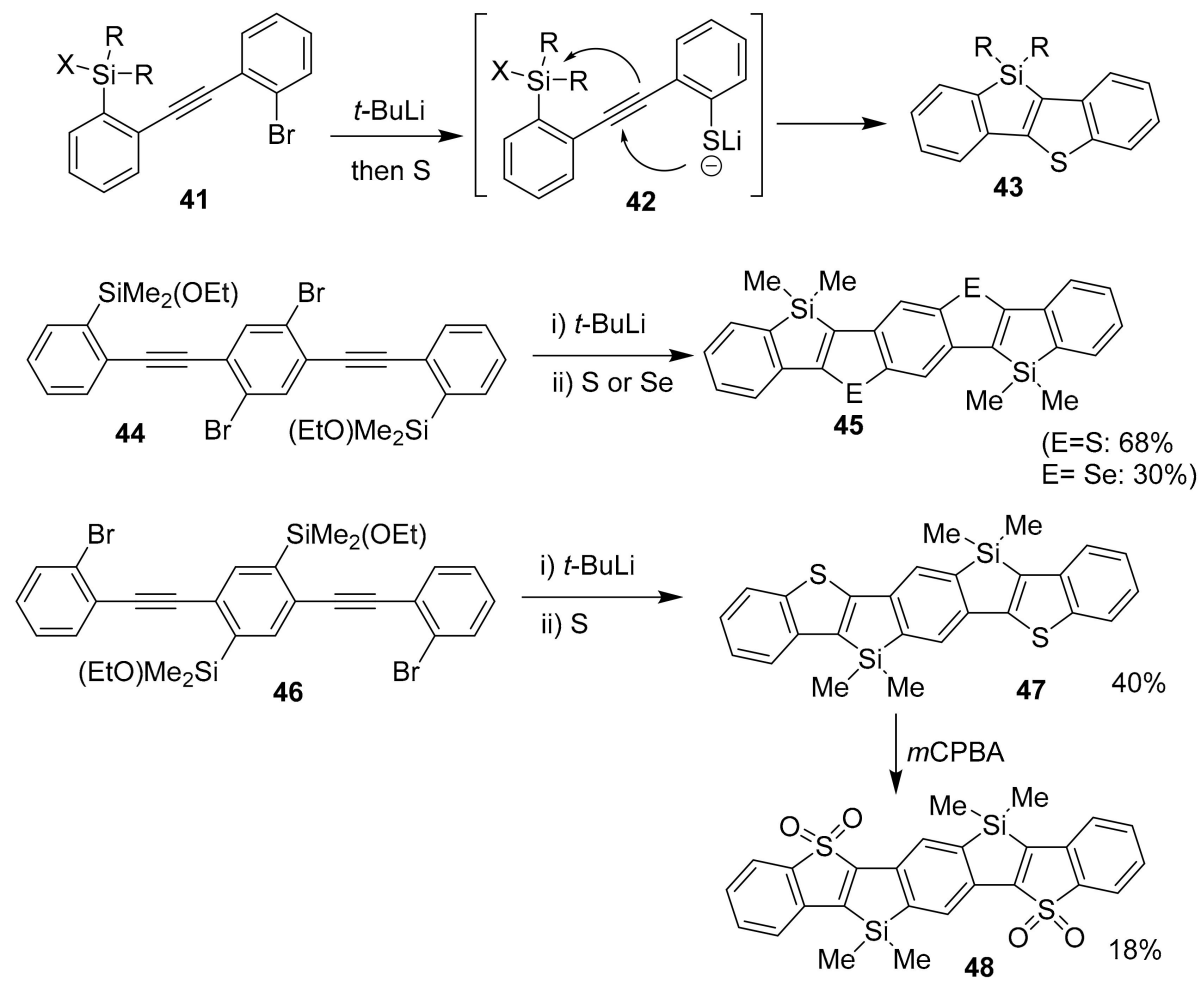
Synthesis of ladder molecules.

In 2007, Yamaguchi and coworkers reported a new cascade anionic double cyclization of (*o*-silylphenyl)(*o*-halophenyl)acetylenes **41** by an initial lithiation followed by adding chalcogen metals (S, Se) giving silicon and chalcogen-bridged stilbenes **45, 47,** and **48** (Scheme 7) [7]. The success of this method to form Si,S-bridged ladder phenylenevinylene structures relied on a new cascade anionic cyclization. Interestingly, the lithiation of **41** formed by the exchange with *t*-BuLi followed by adding elemental sulfur gave a thiolate anion **42**, which further cyclized via a 5-endo-dig process, followed by a nucleophilic substitution at the silicon center to afford the doubly-cyclized Si,S-bridged stilbene **43**. Based on the success of this cyclization, a series of diacetylenic starting materials **44** and **46** have been successfully used to prepare several ladder distyrylbenzenes **45, 47,** and **48** in moderate yields. Along with these syntheses, several interesting results in molecular packing studies and physical properties such as high intensity fluorescence pointed towards their potential applications in organic electronic devices [7].

### 2.2. Metal-Catalyzed [2 + 2 + 2] Cycloadditions

**Scheme 8:**
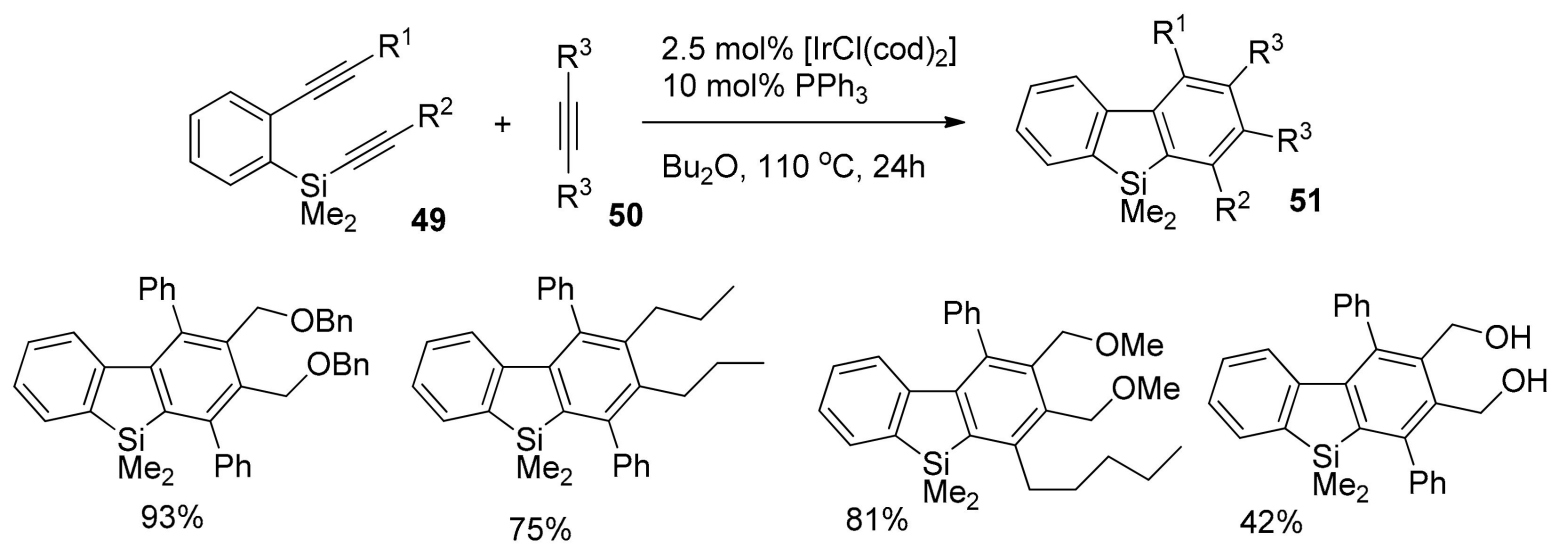
Synthesis of 9-silafluorenes *via* Ir-catalyzed [2 + 2 + 2] cycloaddition.

**Scheme 9:**
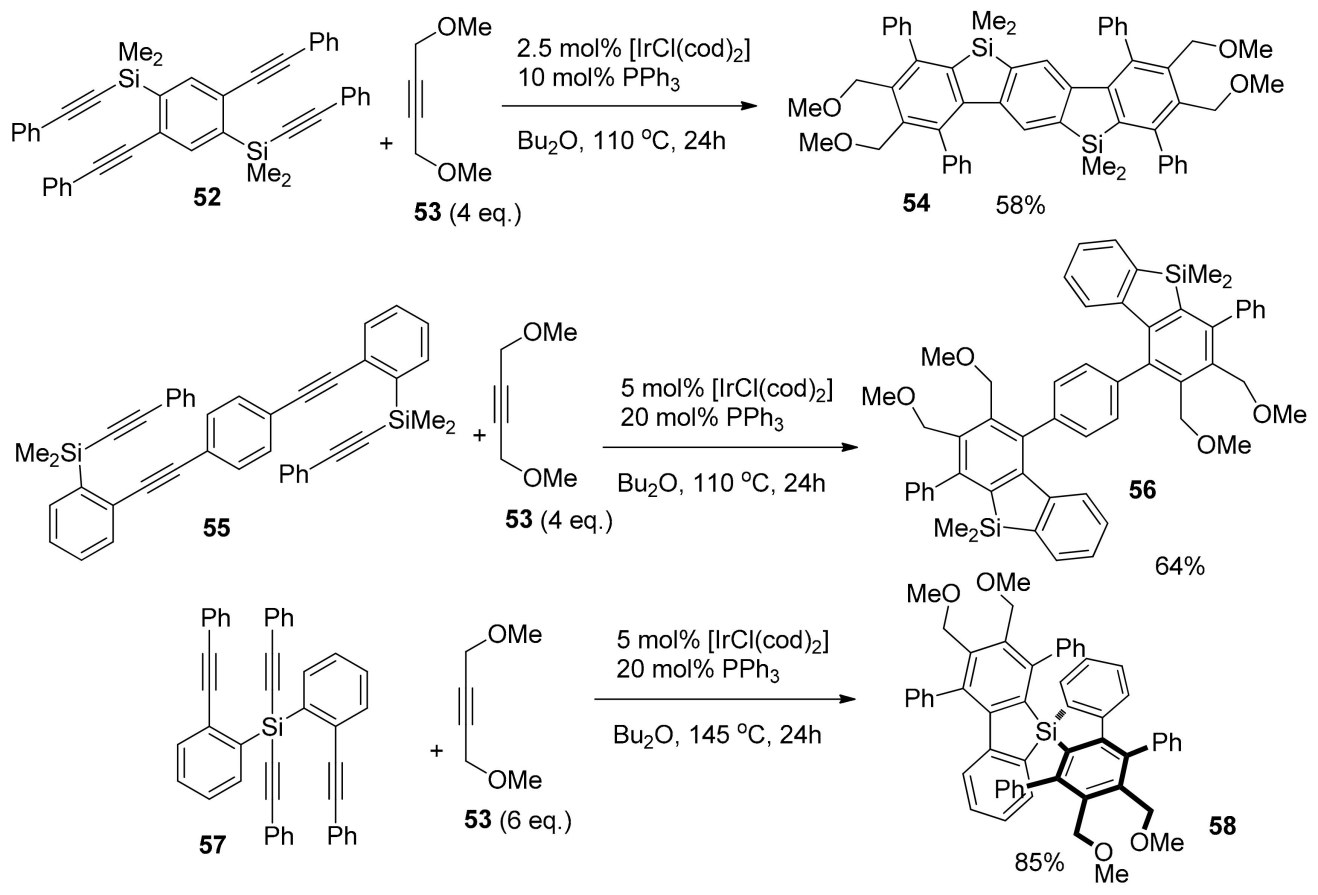
Synthesis of π extended 9-silafluorenes.

Recent development of modern synthetic methods using transition metal catalysts have given efficient pathways to form large π-conjugated polycyclic fused dibenzosilole structures. Thus, there have been many synthetic methods developed for the synthesis of 9-silafluorene and derivatives. Transition metal-catalyzed [2 + 2 + 2] cycloaddition reaction of alkynes to form a benzene ring has been successfully applied in the synthesis of 9-silafluorene and its π–extended derivatives [38]. In 2007, Murakami and coworkers reported the first Ir-catalyzed synthesis of 9-silafluorene derivatives **51** by the [2 + 2 +2] cycloaddition of silicon-bridged 1,6-diynes **49** with alkynes **50** (Scheme 8) [38]. Ladder-type π–extended systems have attracted much attention due to effective conjugation by these rigid coplanar structures. In this study, π–extended 9-silafluorene structures **54, 56, 58** were efficiently synthesized by the [2 + 2 + 2] cycloaddition of tetraynes **52, 55** and **57**, respectively with alkyne **53** (Scheme 9). For instance, this method was efficiently applied to the synthesis of a 9,9′-spiro-9-silabifluorene derivative **58** in 85% isolated yield. Some studies on photophysical and thermal properties of these molecules were performed. Interestingly, molecule **58** exhibited an exceptionally high fluorescent efficiency (91%) [38].

**Scheme 10:**
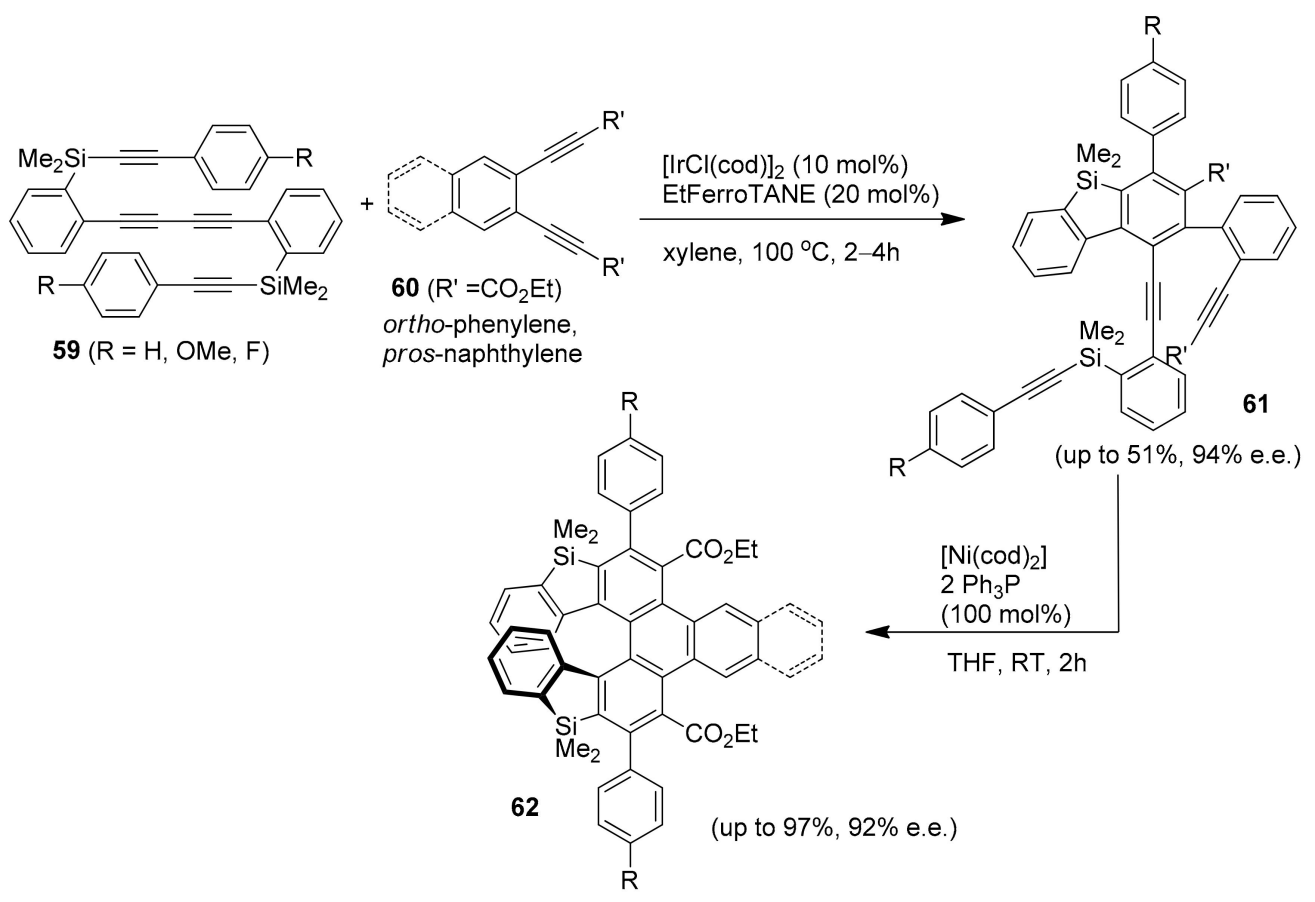
Synthesis of helicene containing 9-silafluorene moiety.

This approach has become one of the most important methods to construct ladder-type π–extended systems containing a 9-silafluorene moiety. Inspired by this pioneering work, the Shibata group has developed an enantioselective synthesis of chiral silahelicenes using Ir-catalyzed [2 + 2 + 2] cycloaddition in the key step (Scheme 10) [39]. In order to get a high yield and enantiomeric excess (e.e.) for this reaction, several chiral bidentate ligands were screened, and (*S*, *S*)-EtFerroTANE ligand in combination with [Ir(cod)Cl]_2_ catalyst gave a cyclized product **61** (up to 51% yield and 94% e.e.) starting from tetrayne **59** and diyne **60**. The subsequent stereospecific intramolecular transformation of chiral triyne **61** did not afford the desired product in satisfying yield while using Ir catalysts. Surprisingly, tandem Ni(cod)_2_/PPh_3_ catalysed cyclizations of chiral triyne intermediates **61** provided target silahelicene **62** in up to 97% yield and 92% e.e. without losing enantiomeric excess.

**Scheme 11:**
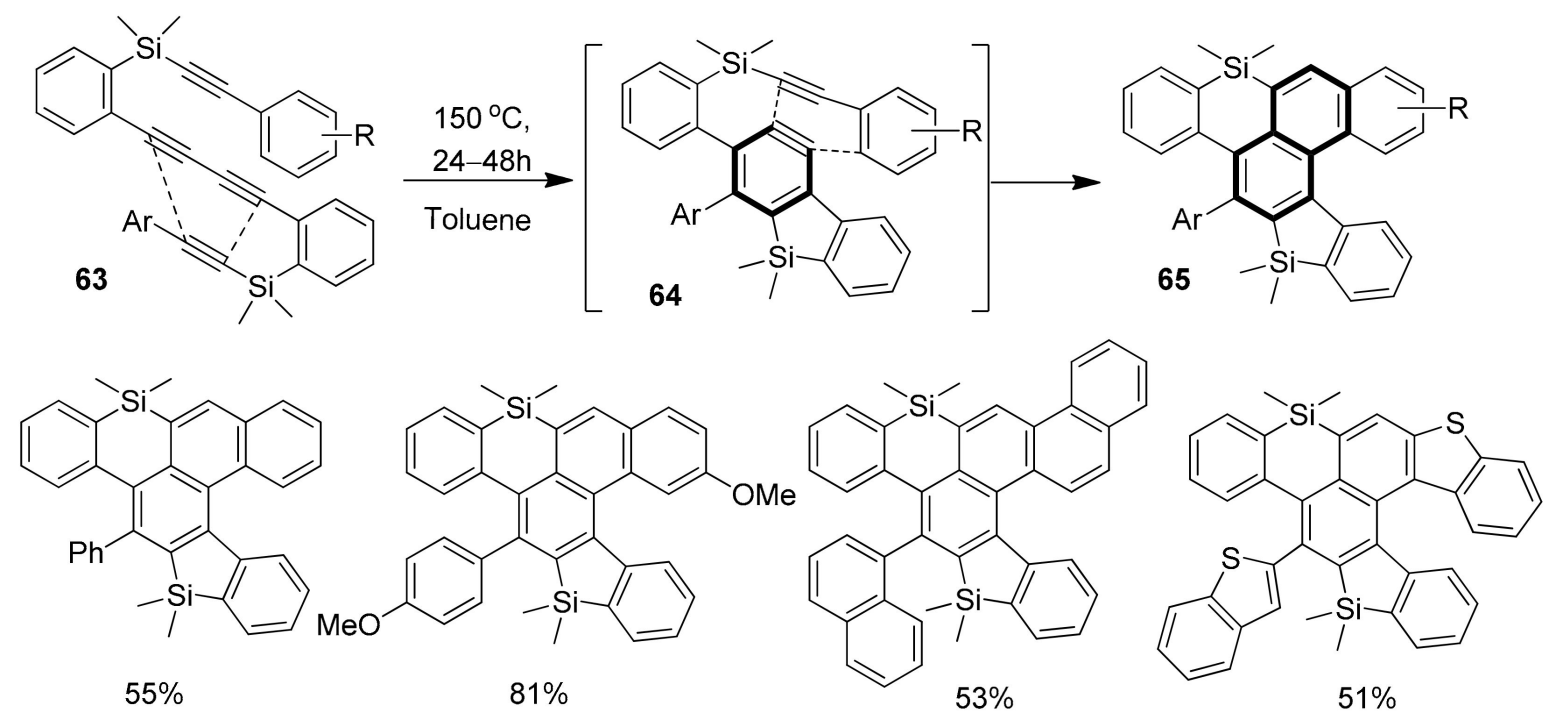
Synthesis of 9-silafluorenes via cycloaddition.

**Scheme 12:**
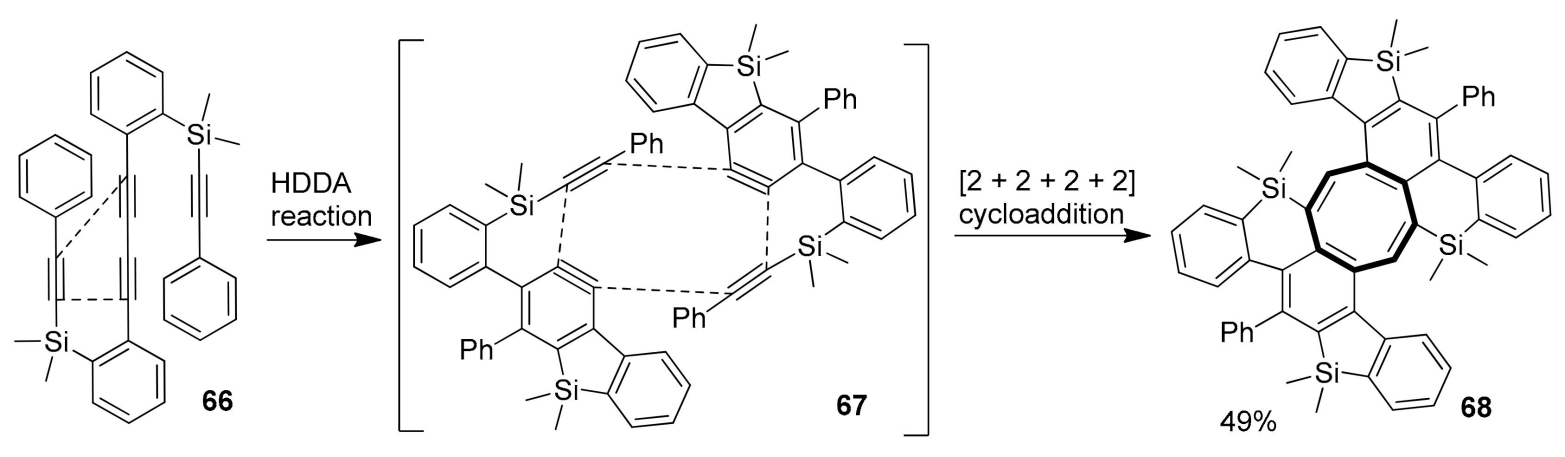
Synthesis of 9-silafluorenes via [2 + 2 + 2 + 2] cycloaddition.

Recently, Shibata et al. have developed the first example of sequential hexadehydro-Diels–Alder (HDDA) and tetradehydro-Diels–Alder (TDDA) reactions using silicon-tethered tetraynes **63** containing a 1,3-diyne moiety via benzosilole-fused benzynes **64** (Scheme 11) [40]. Notably, the silicon-containing polycyclic aromatic system obtained acted as a diene and underwent [4 + 2] cycloaddition with active alkynes. Along with the main product **65**, a side product **68** was isolated and its structure was confirmed by X-ray single crystallography. Remarkably, this undesired side product, a novel eight-membered ring system, was formed via a new thermal [2 + 2 + 2 + 2] cycloaddition of alkynes **67** formed from **66** by cyclotrimerization (Scheme 12). After several optimizations, the yield of **68** was increased to 49% when the reaction was carried out under more concentrated conditions in chlorobenzene solvent.

### 2.3. Transition Metal Catalyzed C-Si Coupling via Cleavage of a C(sp^3^)-Si Bond

**Scheme 13:**
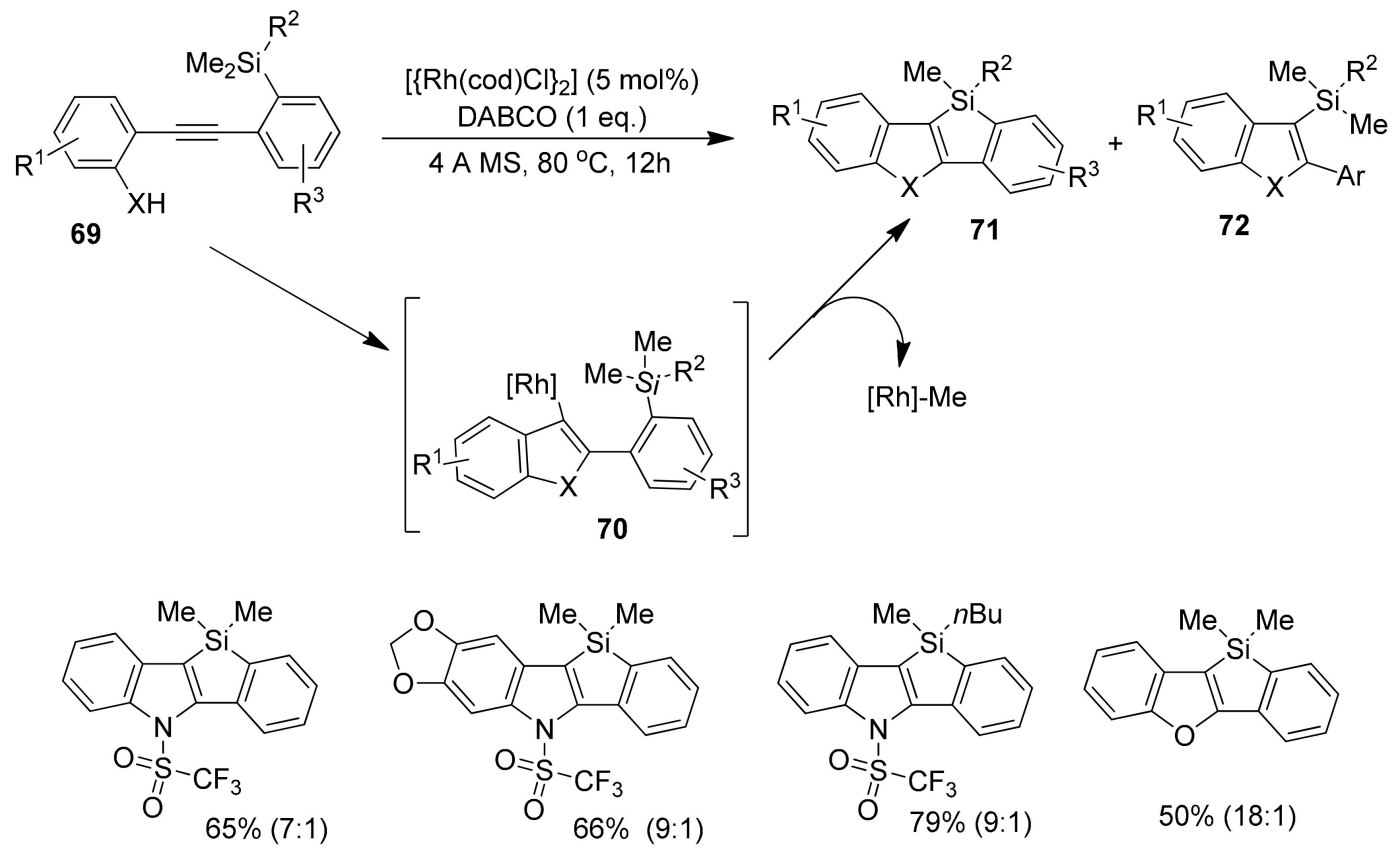
Rh-catalyzed synthesis of heterocycles-fused benzosiloles.

The introduction of an aryl group or a heteroatom into the dibenzosilole ring is instrumental in modifying, and possibly improving the physical properties of the parent (di)benzosiloles. In order to prepare new fused benzosiloles and explore interesting photophysical properties, many synthetic approaches to these motifs have been developed. Among them, recent synthetic methods using transition metals as catalysts in the activation of the Si–C bond have been reported [41]. Especially, Chatani et al. disclosed straightforward Rh-catalyzed Si–C bond activations by transmetalation between arylboronic acids or esters and alkynes. Benzosilole derivatives were prepared in very good yields (up to 98%) [41]. Inspired by this research, He and coworkers developed a new tandem cyclization/Si-C activation process to access heterocycle-fused dibenzosiloles under mild conditions (Scheme 13) [42]. The authors rationalized that the initial intramolecular cyclization of the triflated aniline **69** would form a Rh intermediate **70**, and a subsequent Si–C bond activation would give a cyclized indole-fused benzosilole product **71**. An uncyclized indole side product **72** was also isolated in lower yield. The choice for the trifluoromethanesulfonyl group plays an important role in the success of the cyclization reaction. The reaction was also applied to phenols, affording benzofuro derivatives **71** (X = O) albeit in a lower yield.

**Scheme 14:**
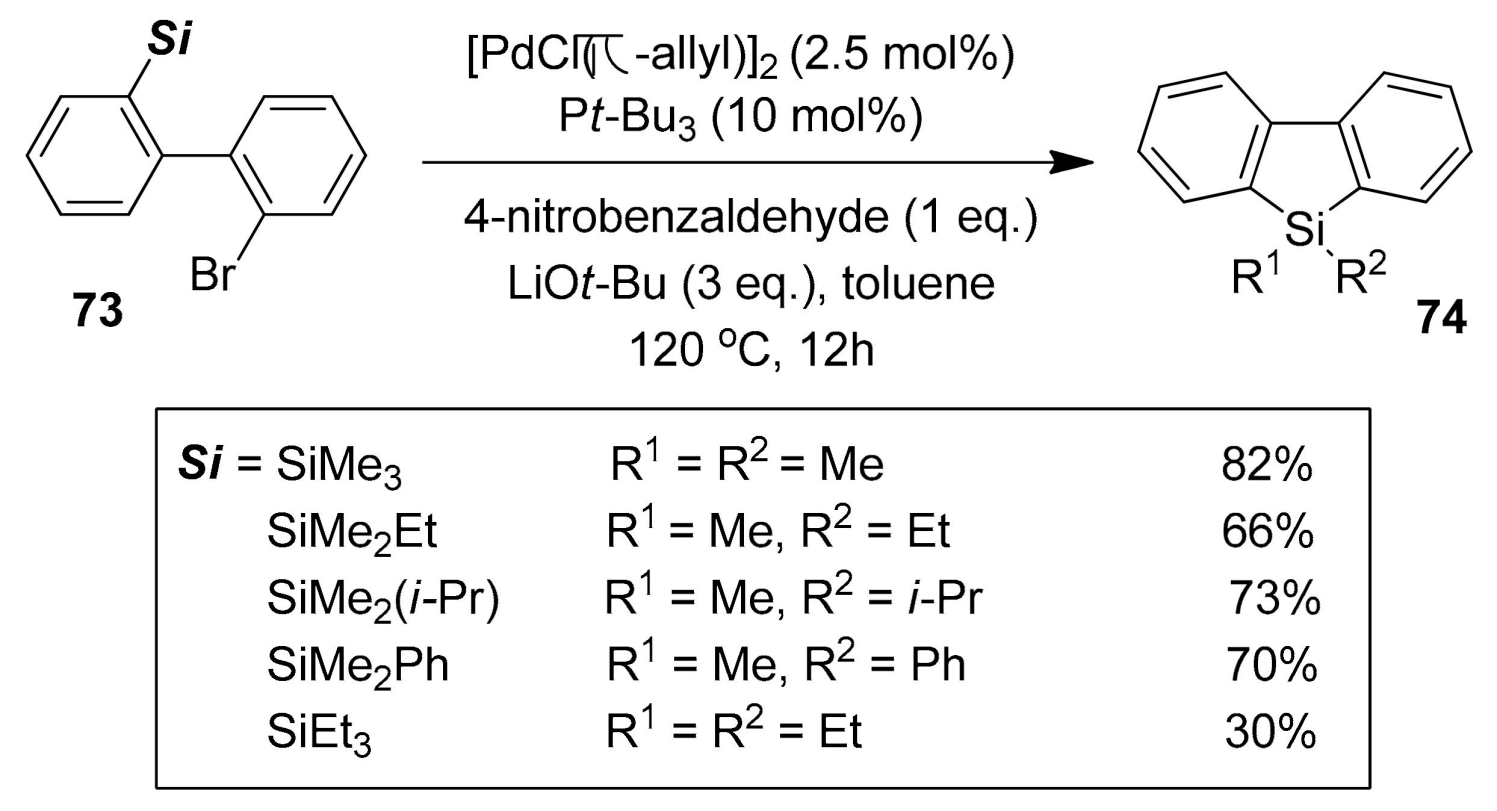
Pd-catalyzed synthesis of 9-silafluorenes.

**Scheme 15:**
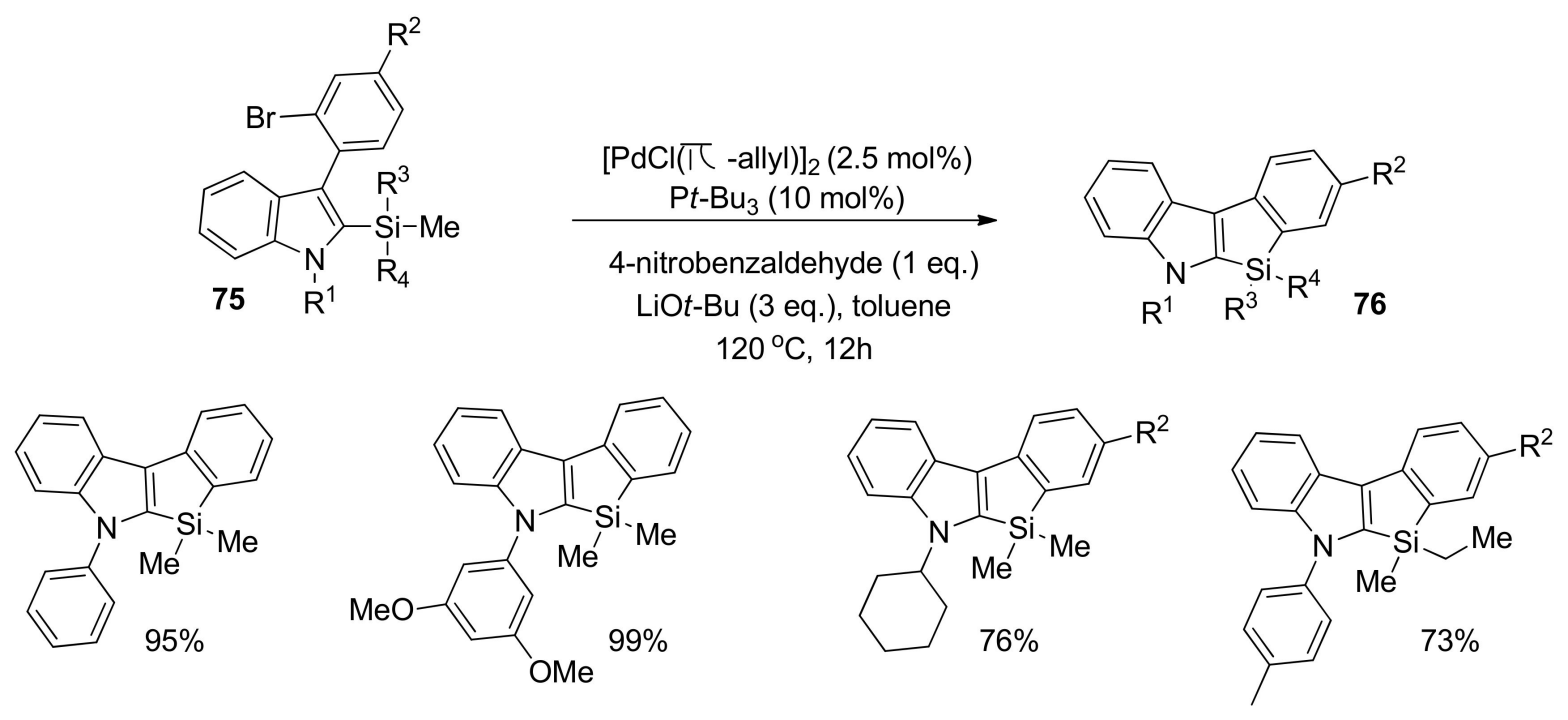
Pd-catalyzed synthesis of heterocycles-fused benzosiloles.

In another approach, Xi et al. demonstrated an efficient Pd-catalyzed synthesis of 9-silafluorenes **74** and benzosilolo[2,3-*b*]indoles **76** via cleavage of a C(sp^3^)–Si bond (of starting materials **73** and **75**) and subsequent intramolecular C(sp^2^)–Si coupling cyclization (Scheme 14 and Scheme 15) [43]. In this transformation, 4-nitrobenzaldehyde **80** plays a key role in promoting the efficiency of the catalytic cycle affording cyclized products in high yields (Scheme 15).

**Scheme 16:**
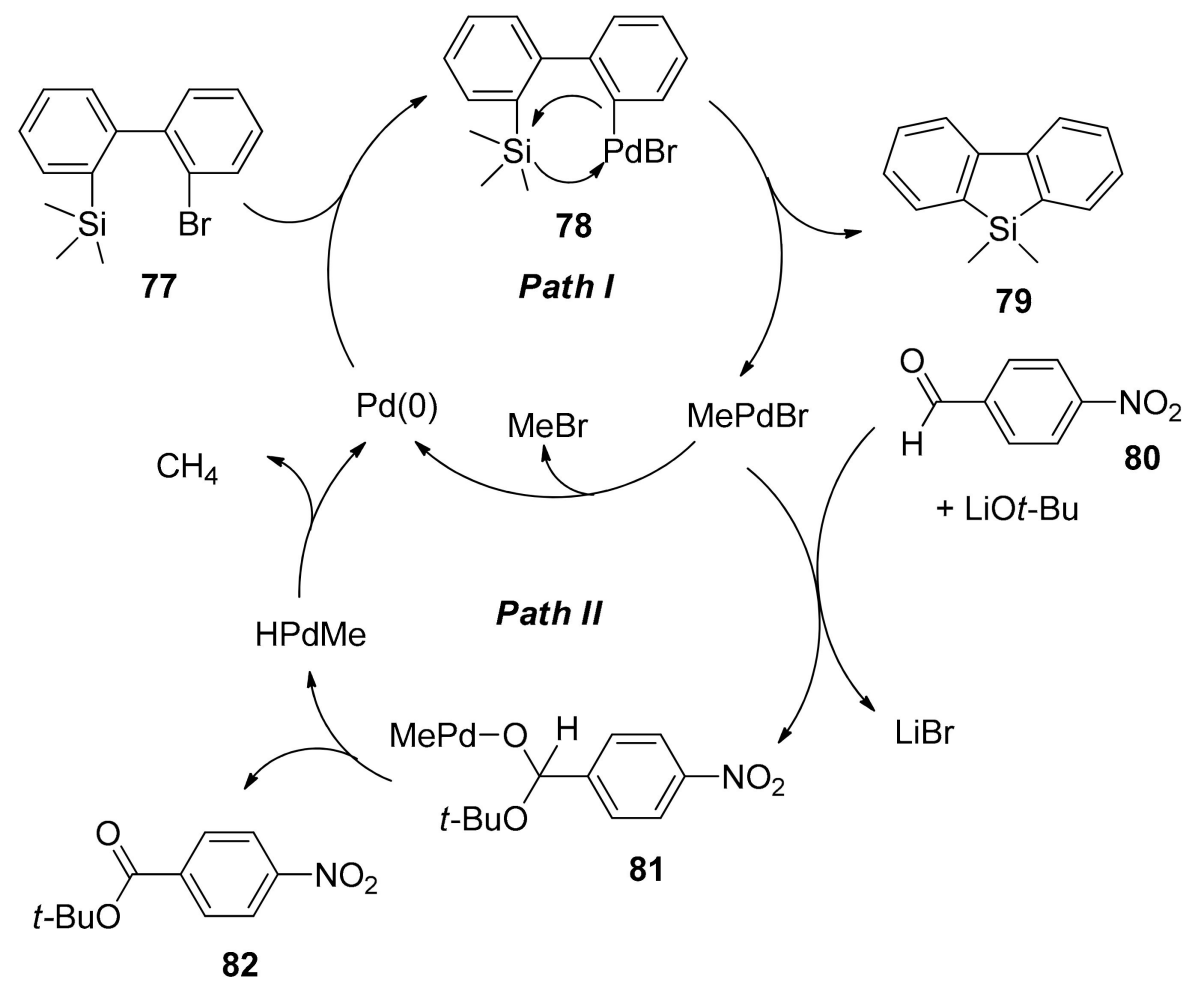
Plausible mechanism for Pd-catalyzed synthesis of 9-silafluorene.

In order to understand the reaction mechanism, several control experiments were carried out. In the absence of 4-nitrobenzaldehyde **80**, MeBr was detected by GC-MS analysis. In the presence of 4-nitrobenzaldehyde **80**, the reaction did not produce MeBr. In fact, the formation of CH_4_ was also confirmed by GC-MS measurement. Significantly, *t*-butyl-4-nitrobenzoate **82** was isolated in 55% yield. Based on these interesting results, a plausible catalytic mechanism for the selective cleavage of CH_3_–Si bond of **77** and subsequent intramolecular C–Si coupling cyclization, involving intermediate **78** and leading to dibenzosilole **79**, was proposed (Scheme 16) [43].

### 2.4. Metal-Catalyzed C–H Activations

**Scheme 17:**
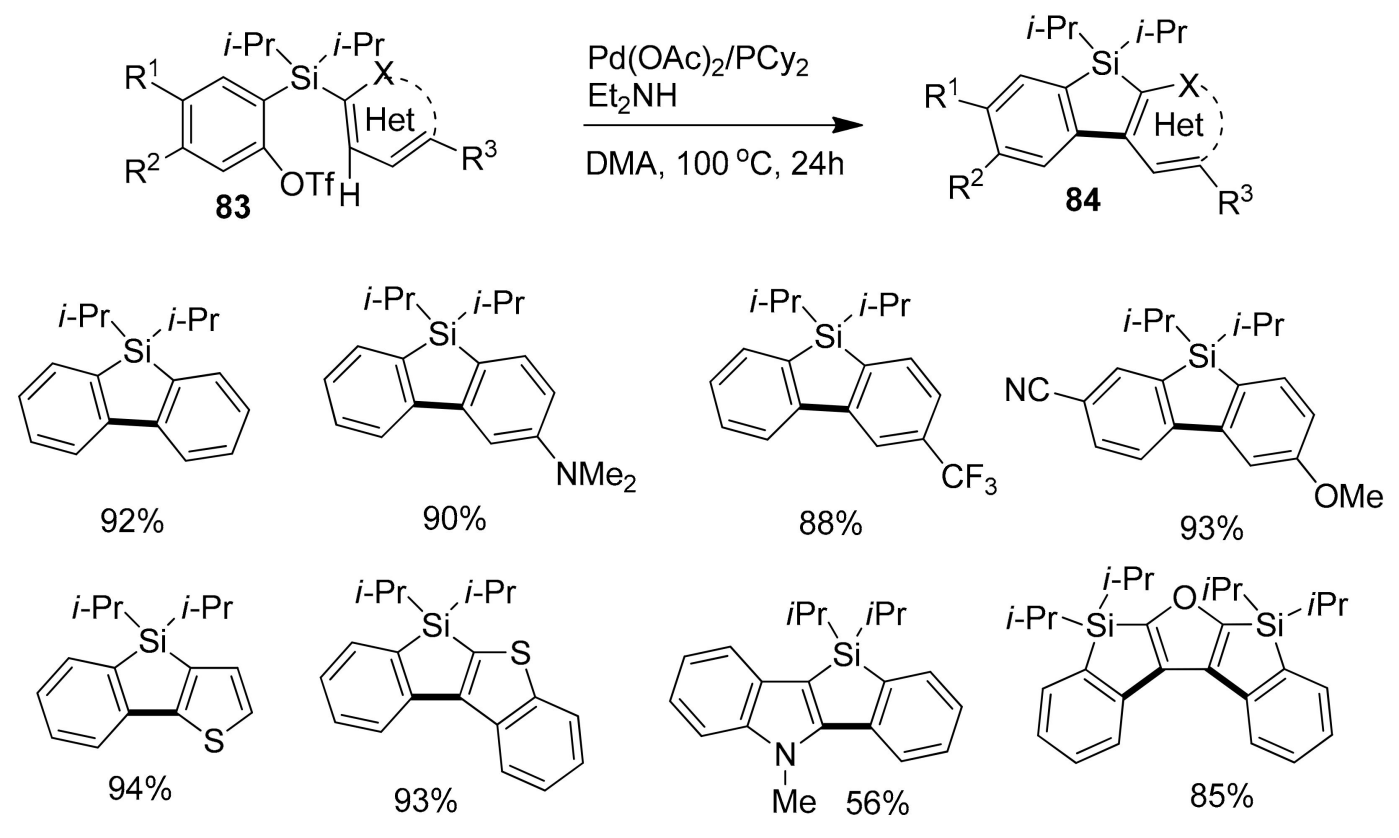
Pd-catalyzed synthesis of 9-silafluorenes and heterocycles-fused benzosiloles.

Transition metal catalyzed arylation of arenes with aryl halides via a C–H activation method has become a very important tool for the construction of aryl-aryl bond formations [21,22,23,24]. Especially, direct Pd-catalyzed intramolecular arylation using silicon-tethering biaryl substrates **83** gave a straightforward pathway to construct heterocycle-fused benzosiloles **84** in very good yields (up to 95%) (Scheme 17) [44,45]. It is important for these Pd-catalyzed intramolecular cyclizations to install bulky substituents on the silicon atom and to employ Et_2_NH base. Furthermore, Shimizu and coworkers realized that these silicon-bridged biaryl molecules showed promising solid state fluorescence. Target compounds exhibiting high solid state luminescent efficiently are currently an important subject of research in the development of OLEDs. Surprisingly, these compounds, when dispersed in a poly(methyl methacrylate) (PMMA) film, showed an intense blue emission with high quantum yield (up to 1.0).

**Scheme 18:**
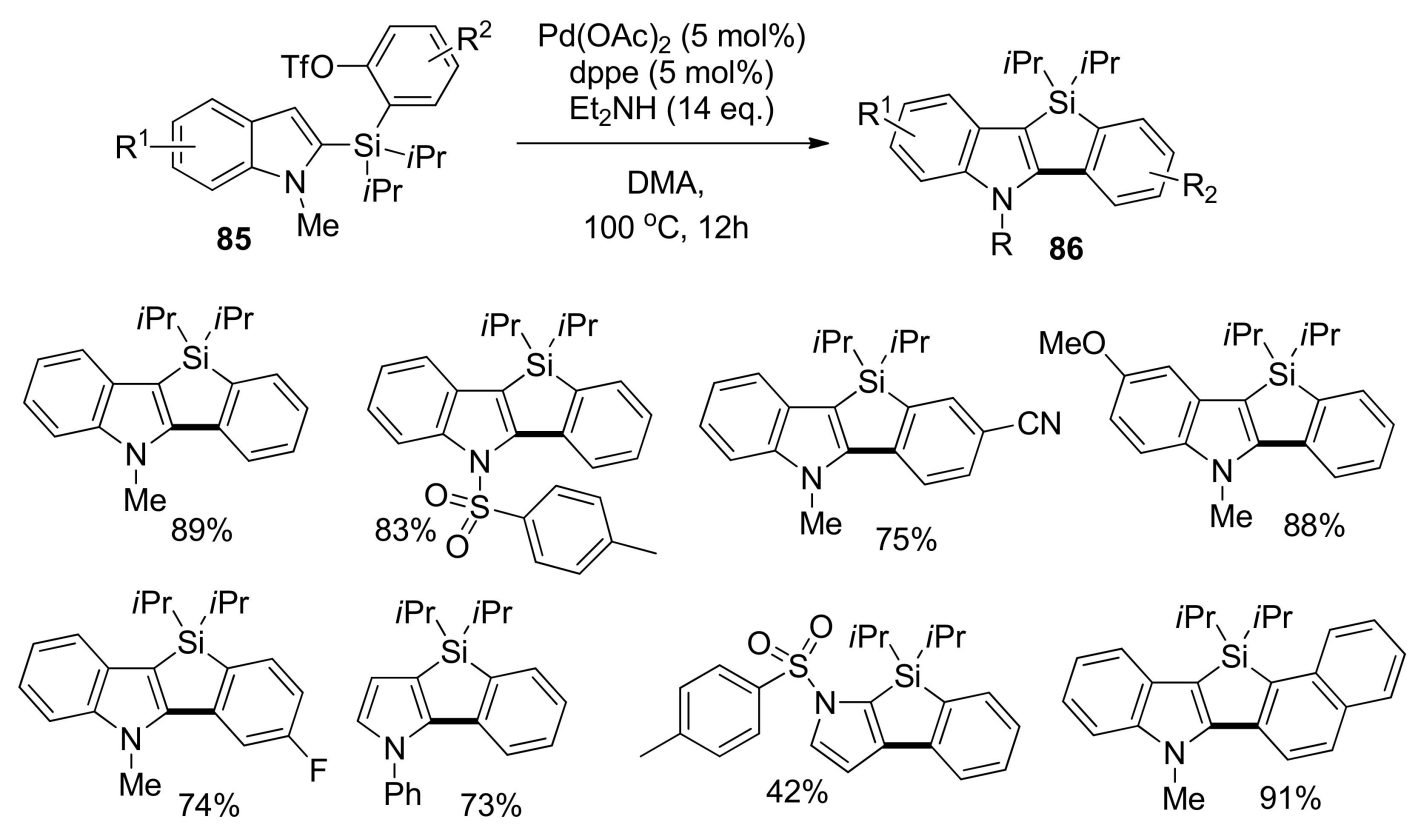
Pd-catalyzed synthesis of rearranged heterocycles-fused benzosiloles.

Based on the success of this research, Shimizu et al. tried to prepare new derivatives of this molecule **84** in order to explore their extraordinary physical properties [46]. Instead of using PCy_3_ as a monodentate ligand, a bidentate ligand (dppe) was used in the combination with Pd(OAc)_2_ catalyst (Scheme 18). Surprisingly, this led to the observation of a new Pd-catalyzed domino process which consisted of an initial intramolecular coupling of **85** followed by a novel rearrangement of the C-Si bond to form indole-fused benzosiloles **86** in moderate to very good yields. Similar to the previous report, all of the prepared compounds showed excellent blue fluorescence with high quantum yields in the solid state.

**Scheme 19:**
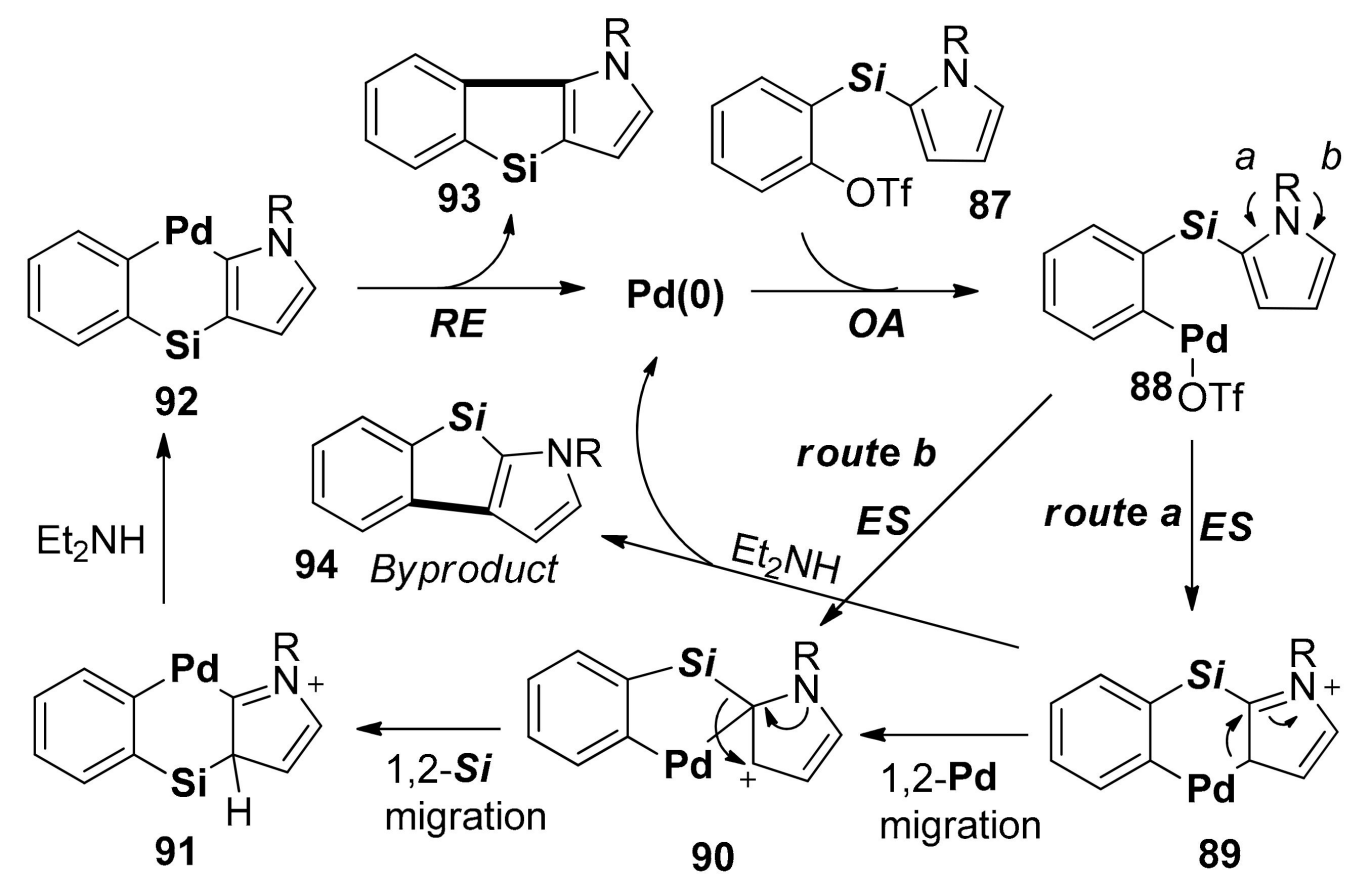
Possible mechanism for Pd-catalyzed synthesis of heterocycles-fused benzosiloles.

A plausible mechanism for the formation of indole-fused benzosiloles was proposed in the Scheme 19 [46]. In general, the first oxidative addition (OA) of Pd catalyst to starting material **87** occurred to afford arylpalladium **88** which could undergo intramolecular electrophilic substitution (ES) to give the palladacycle intermediate **89** (route a), followed by a 1,2-migration step leading to cationic palladacycle **90**. In another pathway, cationic palladacycle **90** could be formed via the direct palladation at the 2-position (route b). During the course of this reaction, a byproduct **94** was often isolated which is valuable evidence for the more plausible route a. Subsequently, 1,2-Si-migration process of cationic palladacycle intermediate **91** followed by deprotonation by an amine and then finally reductive elimination (RE) provided the desired product **93** and regenerated the Pd(0) catalyst for the next catalytic cycle. The formation of the byproduct **94** could be explained by the presence of an EWG in the substrates which may destabilize cationic intermediate **89** and retard the Pd migration step.

### 2.5. C–H Silylations

#### 2.5.1. Transition Metal-Catalyzed C–H Silylations

**Scheme 20:**
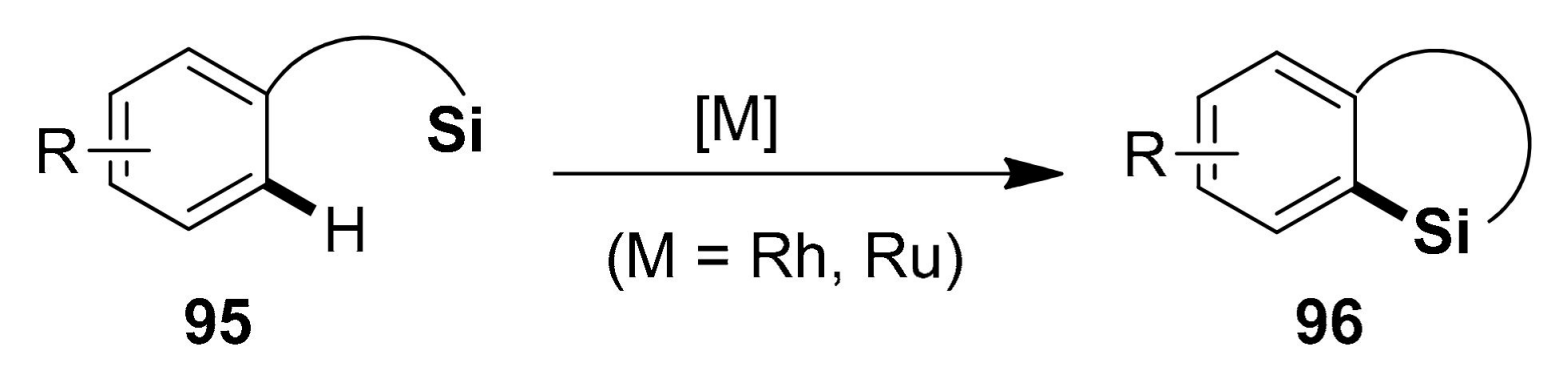
General strategy for transition metal-catalyzed synthesis of fused benzosiloles.

Due to the importance of silicon-containing molecules with potential applications in material science and medicinal chemistry, the formation of a C–Si bond of benzosilole **96** by direct C–H activation of precursor **95** currently is an interesting topic in modern organic synthesis [18,19]. This approach could give a straightforward approach to value-added molecules offering a single step reaction and atom economy in comparison to classic processes. According to the proposed concepts by Cheng and Hartwig [19], this method could be divided into three categories: (*i*) directing-group-assisted intermolecular C–H silylation, (*ii*) undirected intermolecular C–H silylation, and (*iii*) intramolecular C–H silylation (Scheme 20).

**Scheme 21:**
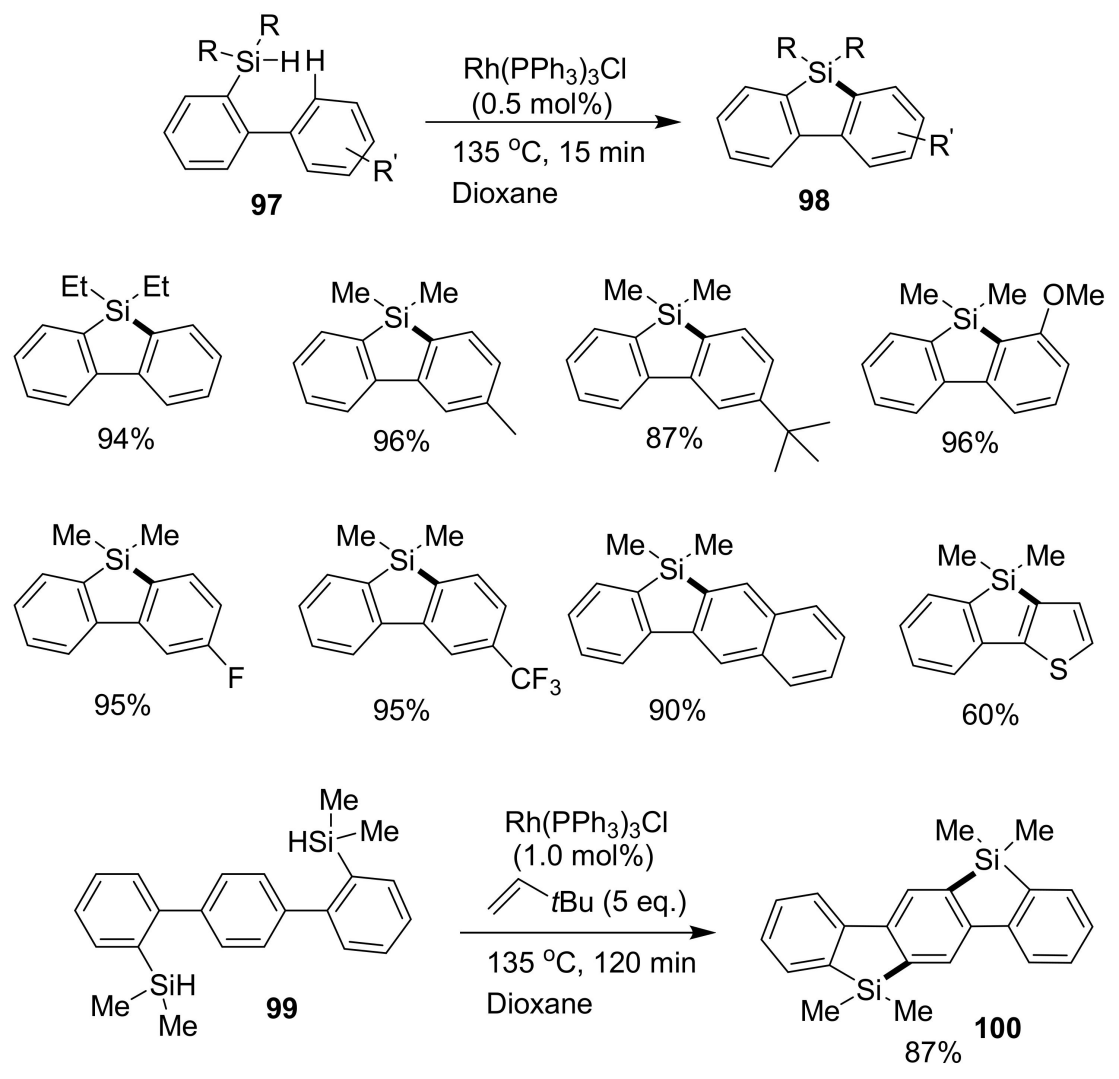
Synthesis of benzosilole derivatives by Rh-catalyzed C-H silylation.

Among these concepts, the process *via* transition metal catalyzed intramolecular C–H arylation is the easiest to be carried out. The Takai group reported the first Rh-catalyzed synthesis of 9-silafluorenes **98** using the cleavage of Si–H and C–H bonds of **97 [47]**. In this process, a new Si–C bond was formed by the activation of both Si–H and C–H via dehydrogenation. Notably, only low catalyst loading (0.5 mol% of Rh(PPh_3_)_3_Cl) and short reaction times (15 min) were required to give high yields of silafluorene derivatives (up to 96%). This method was demonstrated to be very efficient for the synthesis of ladder-type *bis*-silicon-bridged *p*-terphenyl **100** using a double cyclisation starting from **99** (Scheme 21).

**Scheme 22:**
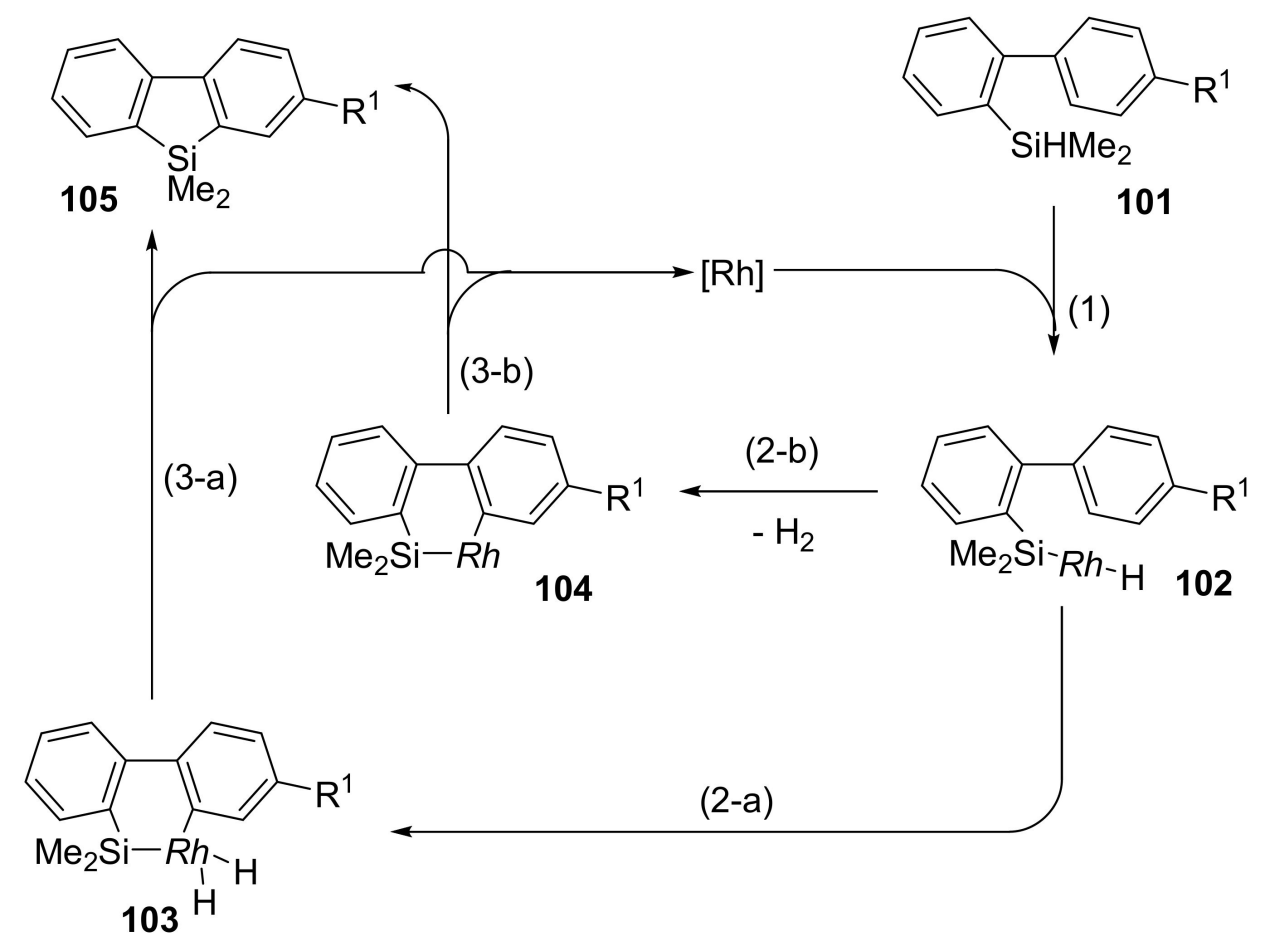
Plausible mechanism for the synthesis of dibenzosilole derivatives via Rh-catalyzed C–H silylation.

Takai and coworkers proposed a possible mechanism (Scheme 22) for the Rh-catalyzed synthesis of 9-silafluorenes **105** which may occur through some steps as following: (*i*) oxidative addition of hydrosilane group to Rh catalyst via Si–H bond activation of **101**; (*ii*) subsequently oxidative addition of aromatic C–H bond to Rh center of **102** via C–H activation (route a or route b); (*iii*) reductive elimination of either 103 or 104 to provide 9-silafluorene products **105** and regenerate the Rh catalyst for the next catalytic cycle [47]. Then, a simple study involving deuterium labelling experiments was performed to understand the rate-determining step for the formation of 9-silafluorenes. This result confirmed that the C–H bond activation of the phenyl group is the rate-determining step.

**Scheme 23:**
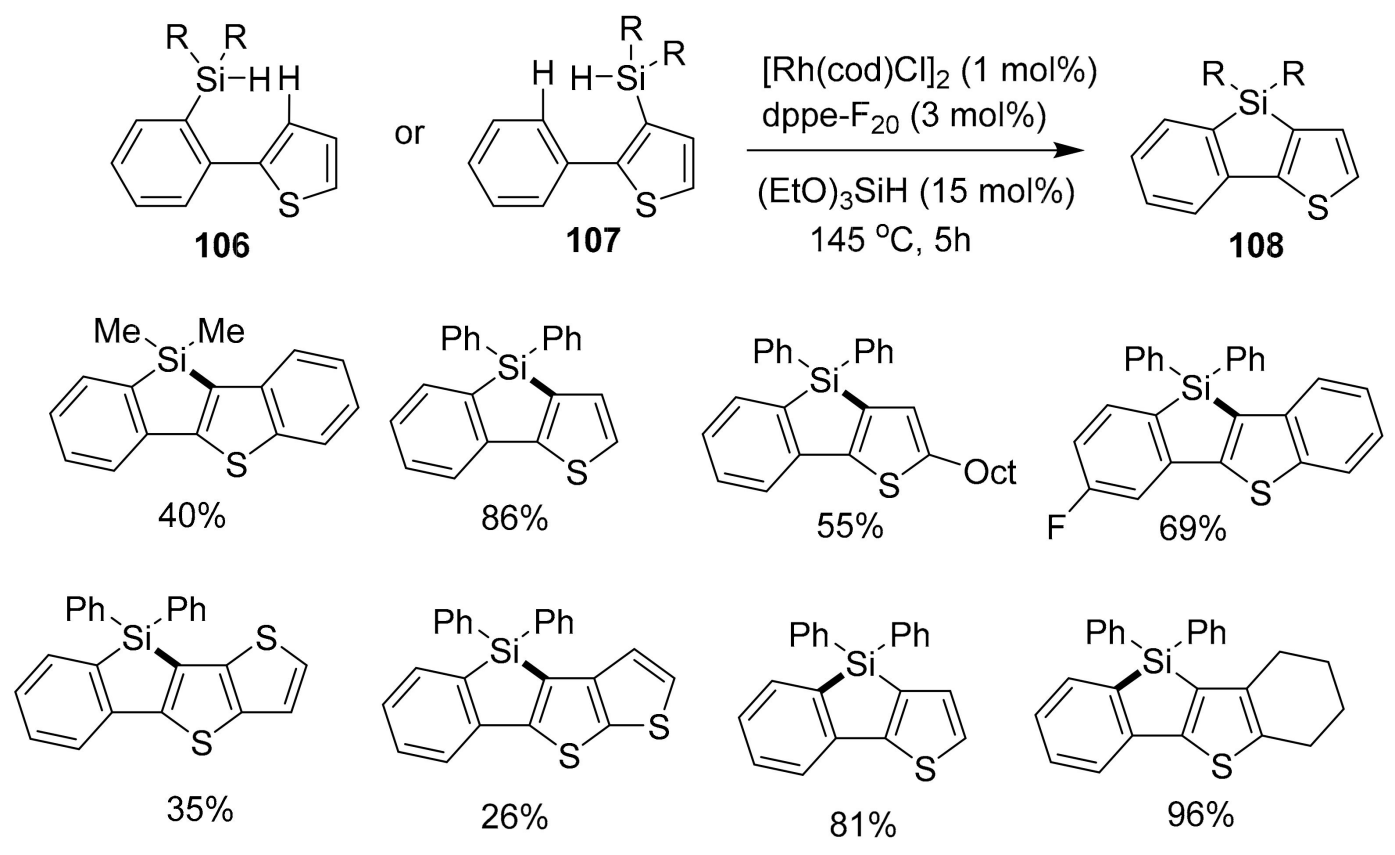
Synthesis of fused benzosilole derivatives by Rh-catalyzed C–H silylation.

**Scheme 24:**
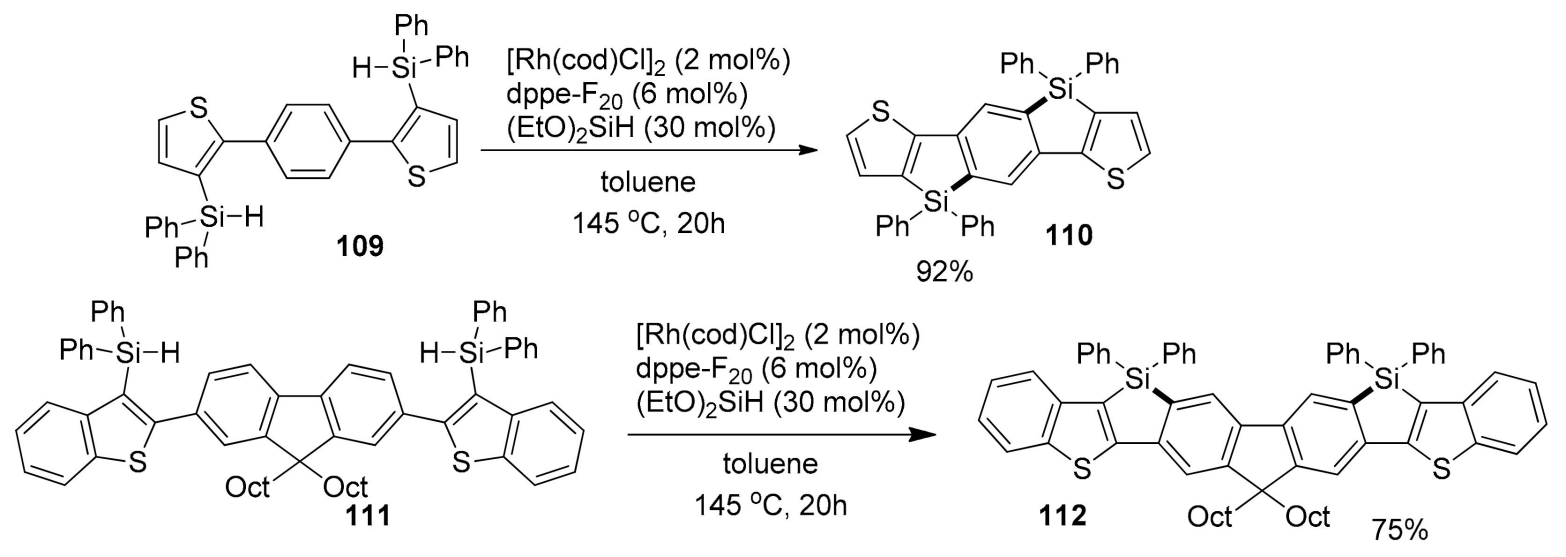
Synthesis of π-extended benzosilole derivatives by two-fold Rh-catalyzed C–H silylations.

In 2017, Mitsudo et al. reported a similar method to prepare π-extended benzosilolothiophenes **108** by Rh-catalyzed dehydrogenative cyclizations of isomeric silanes **106** or **107** (Scheme 23) [48]. Interestingly, a highly electron-deficient ligand (dppe-F_20_) was found to be the most efficient ligand in combination with [Rh(cod)Cl]_2_. This catalytic system was successfully and regioselectively applied for the synthesis of highly π-extended benzosilolothiophenes **110** and **112** in 92% and 75%, respectively starting from **109** and **111**(Scheme 24).

**Scheme 25:**
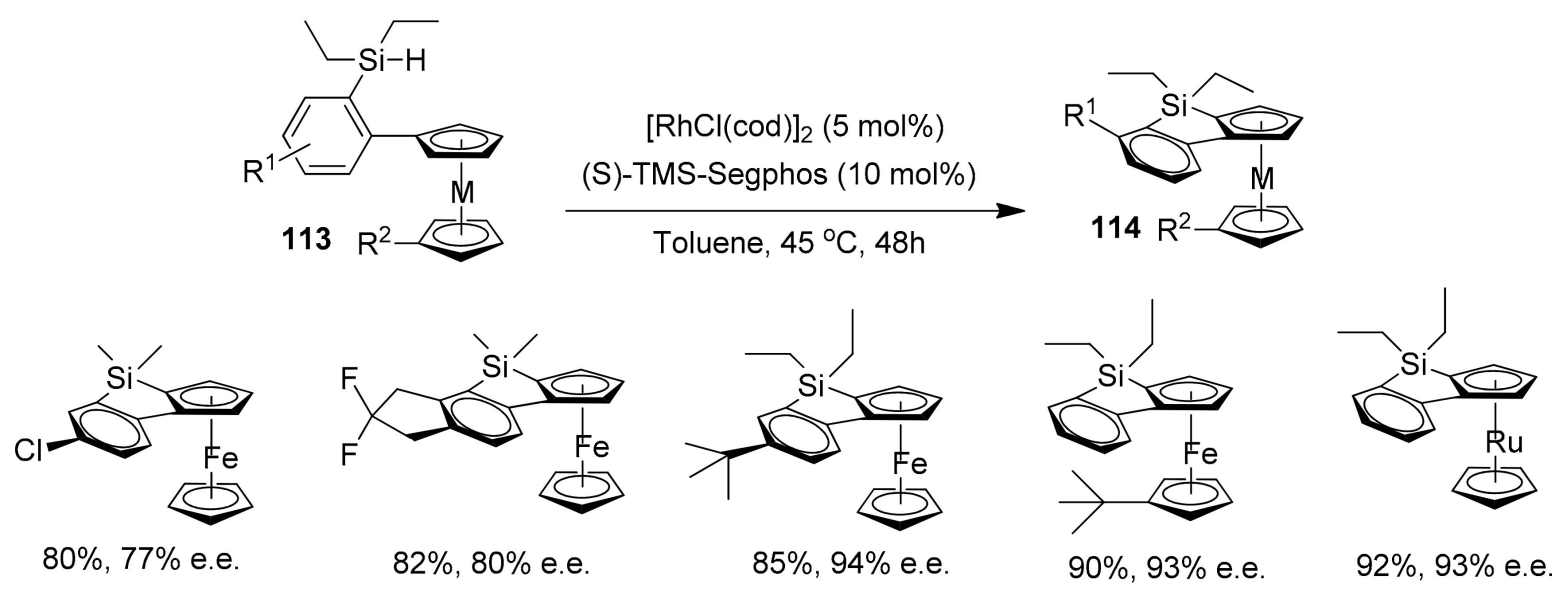
Rh-catalyzed synthesis of benzosilolometallocenes via C–H silylation.

He and coworkers successfully applied this method to silane **113** for an enantioselective Rh-catalyzed synthesis of benzosilolometallocenes **114** (Fe, Ru) (Scheme 25) [49]. In the presence of [Rh(cod)Cl]_2_ catalyst and a chiral bidentate phosphine ligand ((S)-TMS-Segphos), benzosilolometallocenes **114** were prepared in very high yields with e.e. values of up to 99% under mild conditions. In the same year, Murai, Takai and Shibata groups reported very similar research describing the synthesis of benzosilolometallocenes [50,51].

**Scheme 26:**
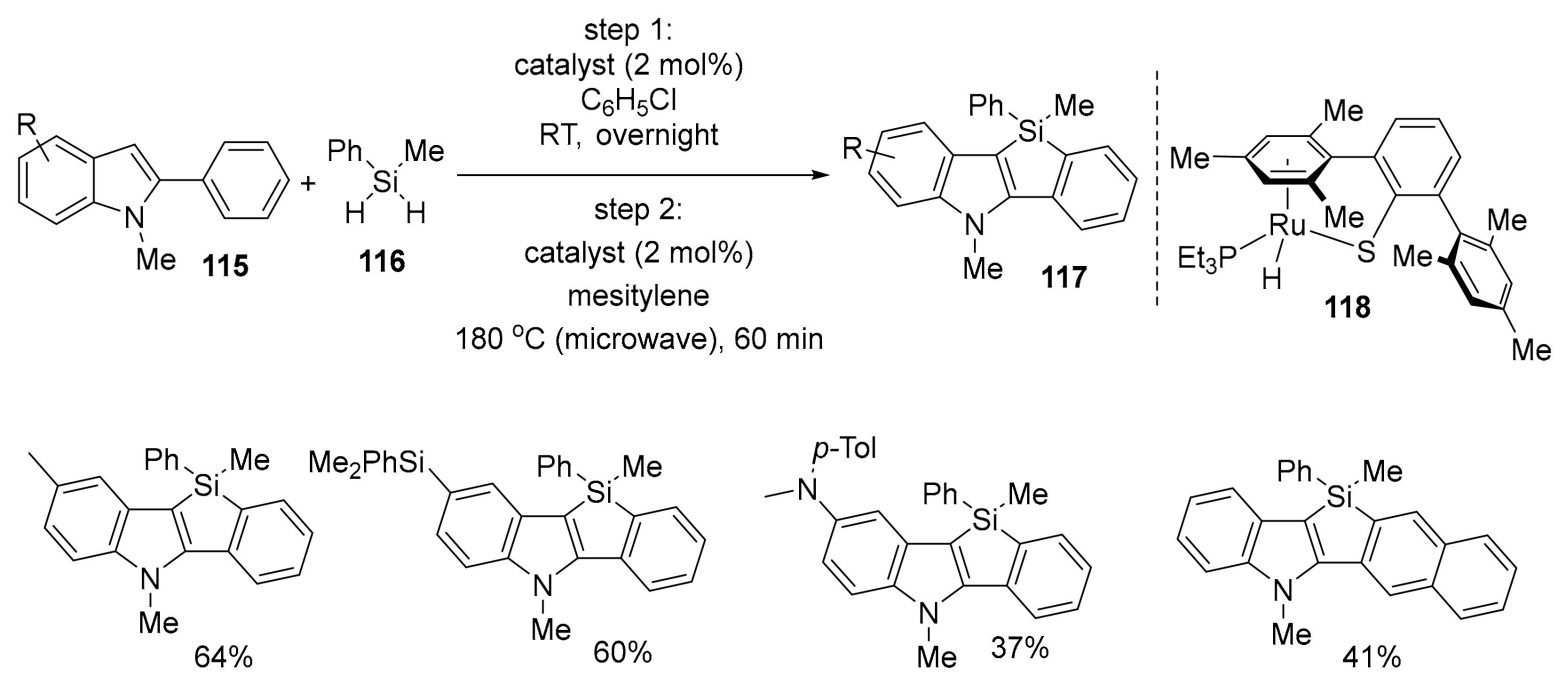
Direct Ru-catalyzed synthesis of indole-fused benzosiloles using dihydrosilanes.

Indole-fused benzosiloles exhibit promising physical properties which can be instrumental in the development of advanced luminescent materials. Therefore, the efficient synthesis of indole-fused benzosiloles derivatives from simple and inexpensive starting materials is highly important. In 2016, Omann and Oestreich reported the direct Ru-catalyzed synthesis of indole-fused benzosiloles **117** by two-fold electrophilic C–H silylation of 2-aryl indoles **115** with dihydrosilanes **116** (Scheme 26) [52]. This transformation is a challenging problem due to the intermolecular dehydrogenation coupling of indole **115** and dihydrosilane **116** being more difficult than the intramolecular dehydrogenation coupling approach. Despite having some limitations in substrate scope, this method is a highly practical approach to the preparation of indole-fused benzosiloles **117** using a Ru catalyst less expensive in comparison to other Rh-catalyzed methods.

**Scheme 27:**
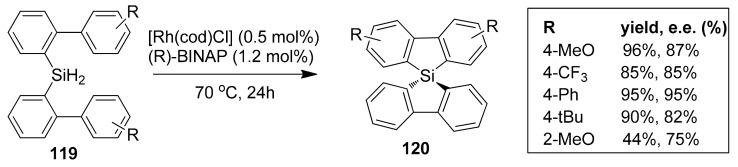
Rh-catalyzed enantioselective synthesis of chiral spiro-9-silabifluorenes.

**Scheme 28:**
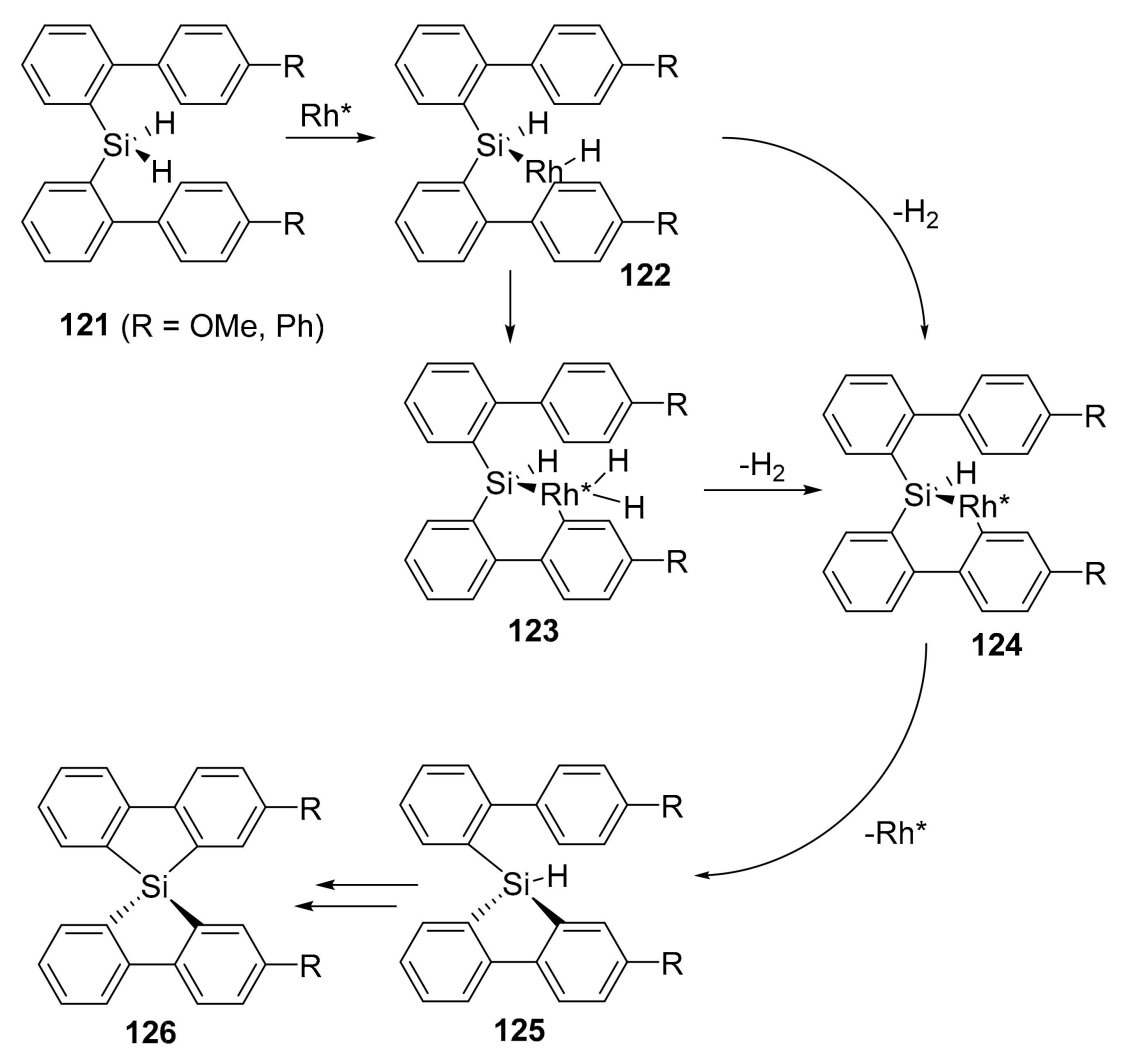
Proposed mechanism for Rh-catalyzed synthesis of chiral spiro-9-silabifluorenes.

As mentioned above, spiro-9-silabifluorene derivatives are promising structures for their use in electronic devices. Thus far, the conventional method using BuLi induced metal exchange only gives a racemic mixture of both enantiomers in the case of R-substituted derivatives. Therefore, the development of new methods to access chiral spiro-9-silabifluorenes **120** is very important for exploring the new class of optically active silicon materials. In 2016, Takai and coworkers described an interesting Rh-catalyzed synthesis of chiral spiro-9-silabifluorenes **120** by dehydrogenative silylation of bis(biphenyl)silane **119** (Scheme 27) [53]. Chiral spiro-9-silabifluorenes **120** were successfully prepared (up to 96% yield and 95% e.e.) under mild conditions. Interestingly, some mechanistic studies were carried out to understand the construction of these tetraorganosilicon stereocenters. A similar mechanism for the synthesis of spiro-9-silabifluorenes (involving compounds **121**–**126**) as mentioned in Scheme 28 was proposed.

**Scheme 29:**
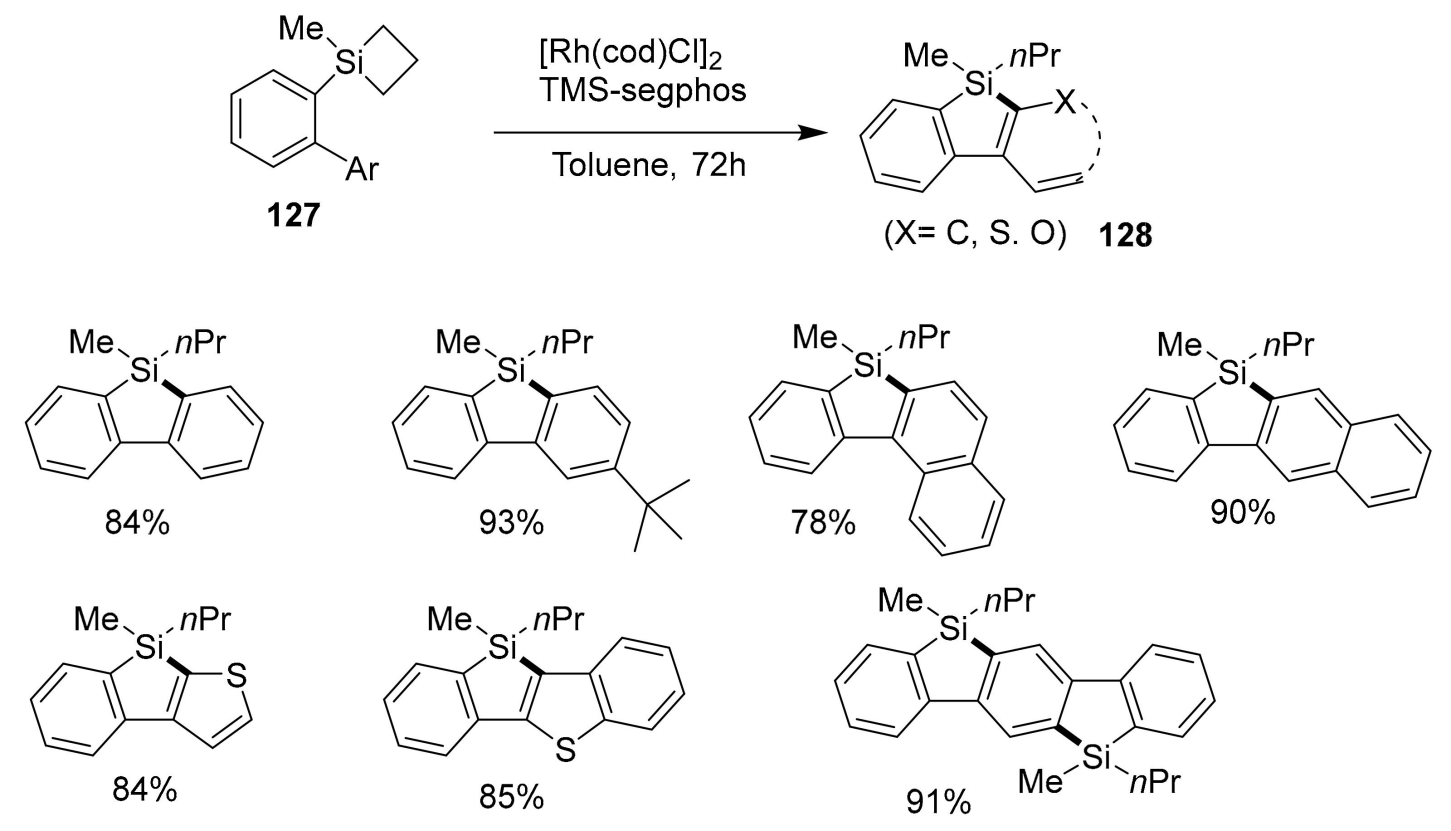
Rh-catalyzed synthesis of 9-silabifluorenes.

**Scheme 30:**
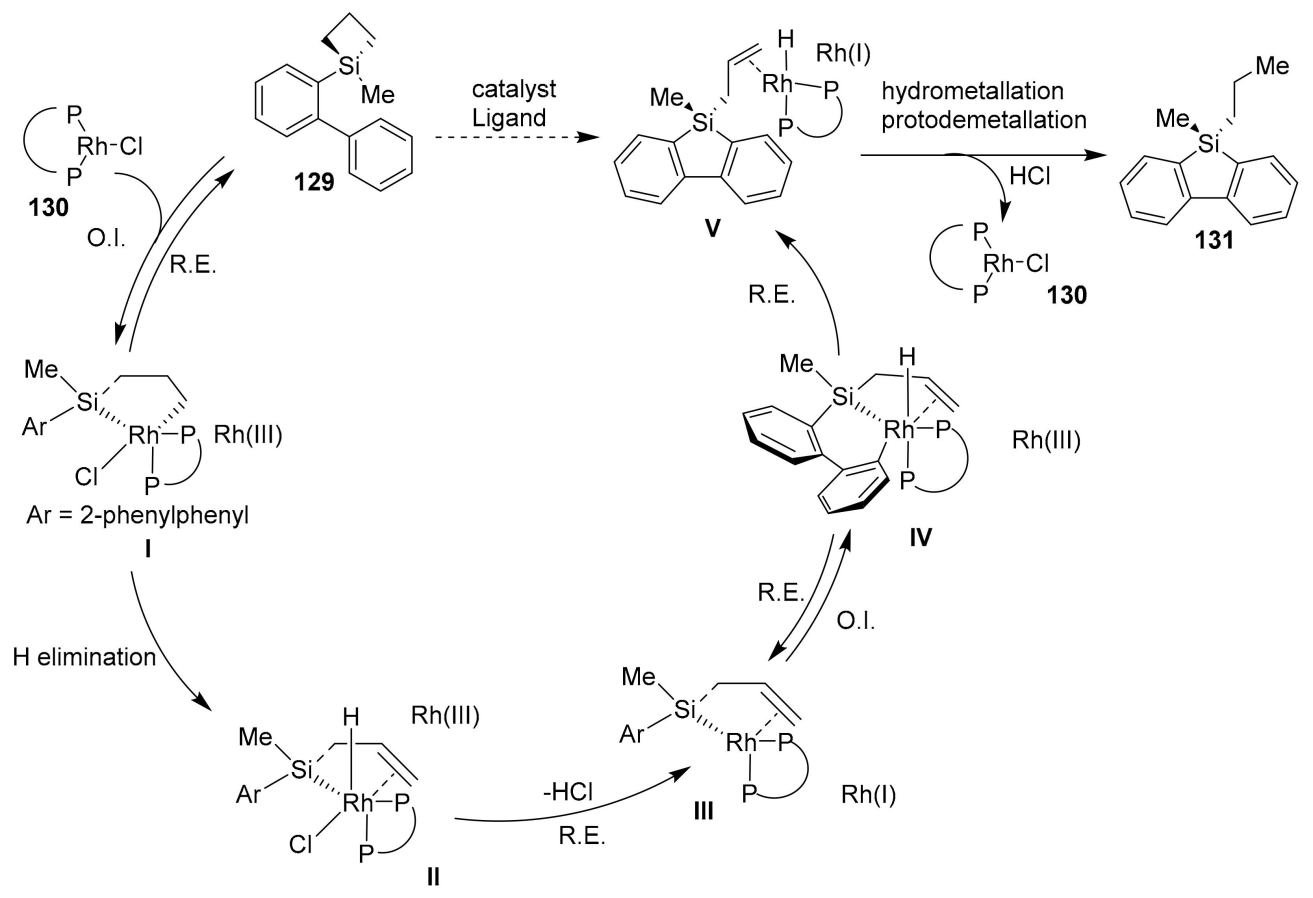
Plausible mechanism for Rh-catalyzed synthesis of 9-silabifluorene.

He and coworkers reported a new method to construct 9-silafluorene derivatives via Rh-catalyzed intramolecular C–H silylation using silacyclobutanes **127** as key starting materials [54]. In the presence of [Rh(cod)Cl]_2_/TMS-segphos catalyst, silacyclobutane **127** underwent sequential C–Si/C–H activations, giving the desired 9-silafluorenes **128** in very good isolated yields (Scheme 29). The proposed catalytic cycle involves a endocyclic *β*-hydride elimination of five-membered metallacycles, which after reductive elimination formed a Si-Rh(I) catalytic active species that is a key intermediate for the next C (sp^2^) –H activation step (Scheme 30).

Based on some experimental results, a plausible catalytic mechanism was proposed in Scheme 30. Firstly, a reversible oxidative insertion (O.I.) of Rh(I) catalyst **130** into silacyclobutane **129** occurred to give five-membered silametallacycle **I** which underwent *β*-H elimination to form intermediate **II**. The reductive elimination step of the Rh(III) complex is believed to provide Rh(I) intermediate **I** along with HCl. Subsequently, Rh(III) intermediate **IV** was formed via a reversible C–H activation. Then, the hydrometallation of terminal alkene **V** and protodemetallation with HCl occurred to give 9-silafluorene product **131** and regenerate the Rh catalyst **130** [54].

**Scheme 31:**
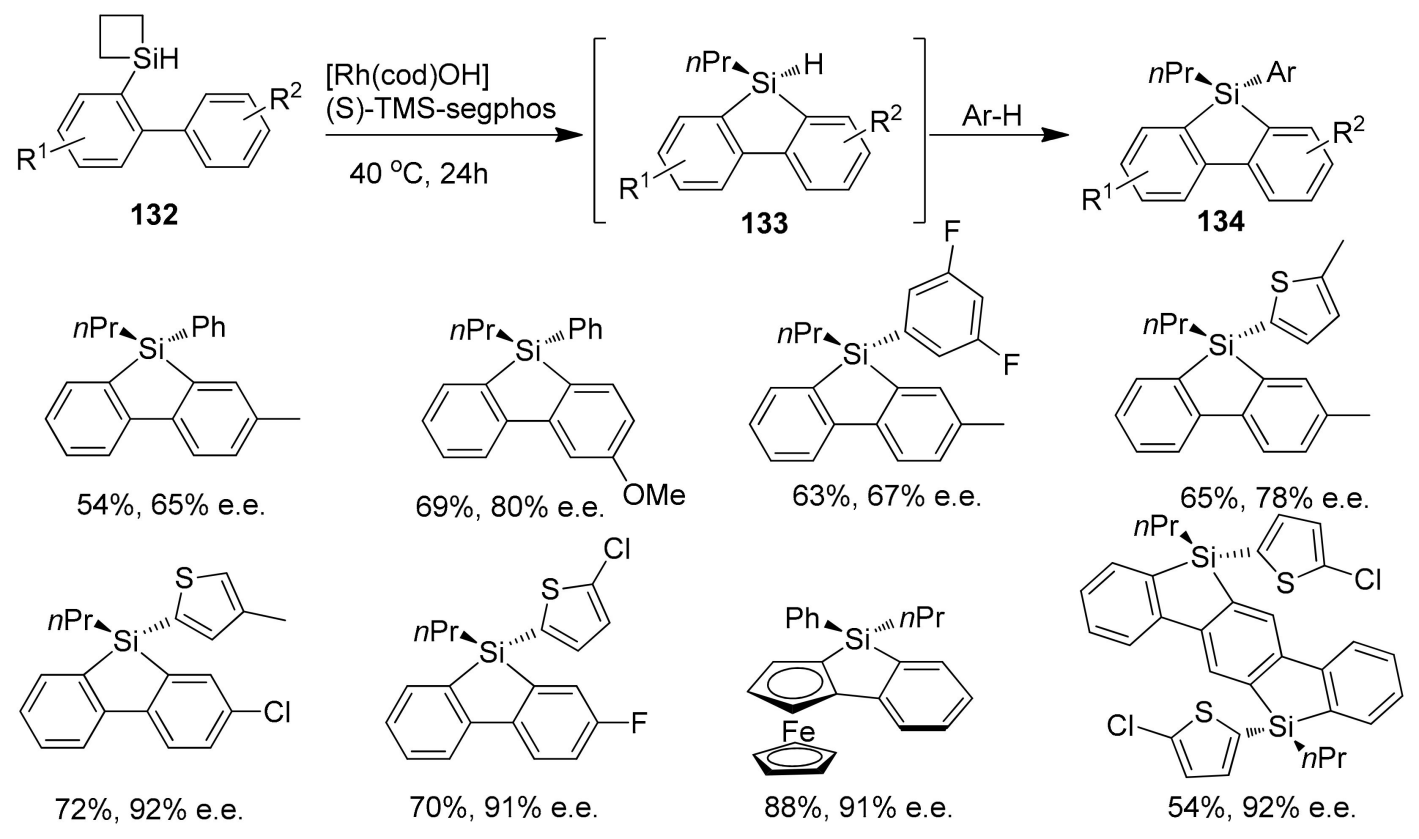
Enantioselective Rh-catalyzed synthesis of 9-silabifluorenes.

Relying on this research, the He group continued developing a useful method to prepare chiral dibenzosiloles **134** via tandem Rh catalyzed desymmetrization and intermolecular dehydrogenative silylations under mild conditions (Scheme 31) [55]. In fact, chiral TMS-segphos was found to be the most suitable bidentate ligand to achieve chiral products in high yields and enantioselectivity up to 93%. In order to understand the mechanism of this transformation, several control experiments were carried out. The stereo-determining step is the silacyclobutane opening/intramolecular C–H silylation step of **132**, giving a chiral silane intermediate **133** which reacted further with arenes.

#### 2.5.2. Synthesis of Dibenzosilole Derivatives via a sila-Friedel-Crafts Reaction

**Scheme 32:**
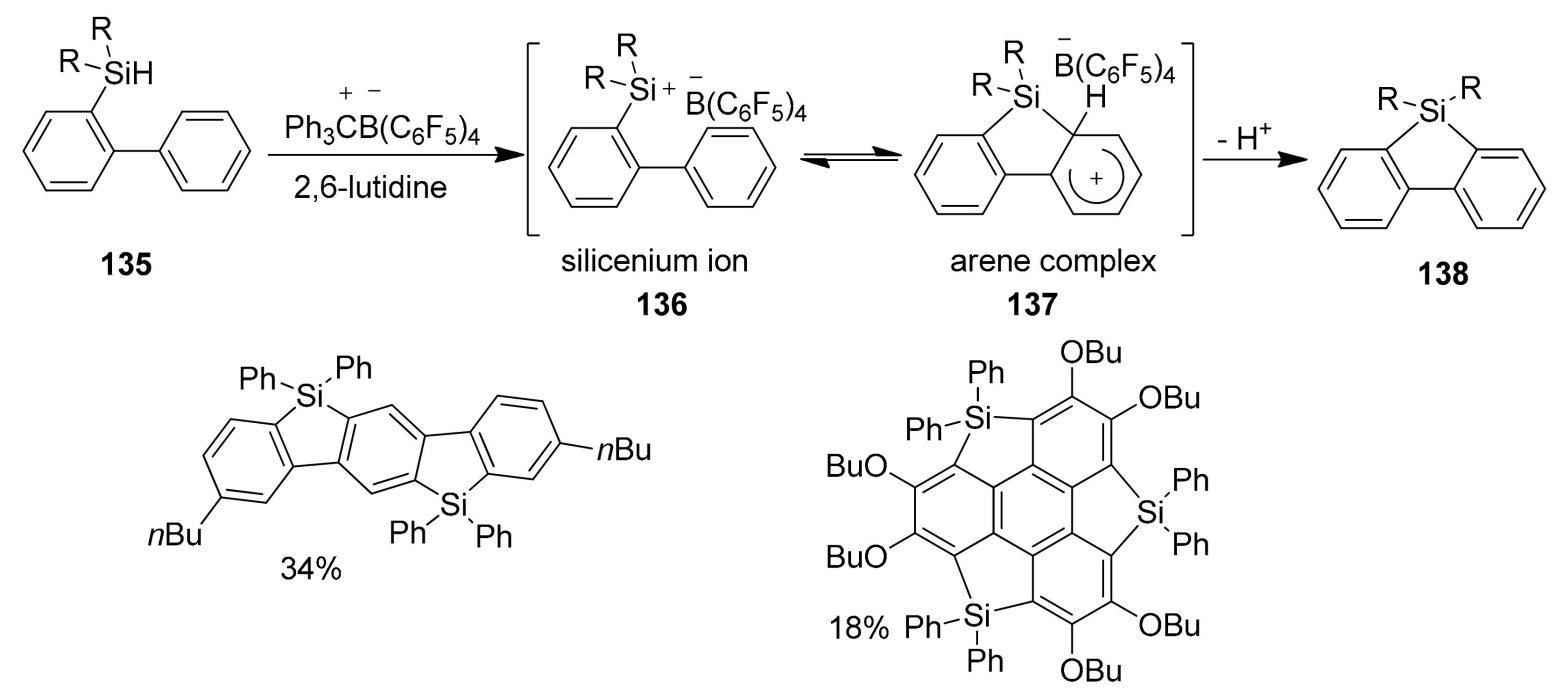
Synthesis of dibenzosilole derivatives via a sila-Friedel-Crafts reaction.

As mentioned above, the drawback of the transition metal-catalyzed C–H silylations is the use of very expensive catalysts (Rh, Ru) and ligands. Therefore, the development of new synthetic methods leading to dibenzosiloles **138** using less expensive metals and/or cheap reagents has been receiving much attention [20]. The Friedel-Crafts reaction is a well-known method for the direct introduction of alkyl substituents into the aromatic rings. However, the sila-Friedel-Crafts reaction of **135** via silicenium ion **136** and arenium ion **137** as intermediates is quite rare and only applicable to electron rich aromatic systems such as ferrocene and pyrroles [56,57]. Remarkably, Kawashima et al. successfully applied an intramolecular sila-Friedel-Crafts reaction to the synthesis of a ladder-type silafluorene and trisilasumanene (Scheme 32). Several studies on the physical properties of these products showed a blue fluorescence in DCM and in the solid state [58].

**Scheme 33:**
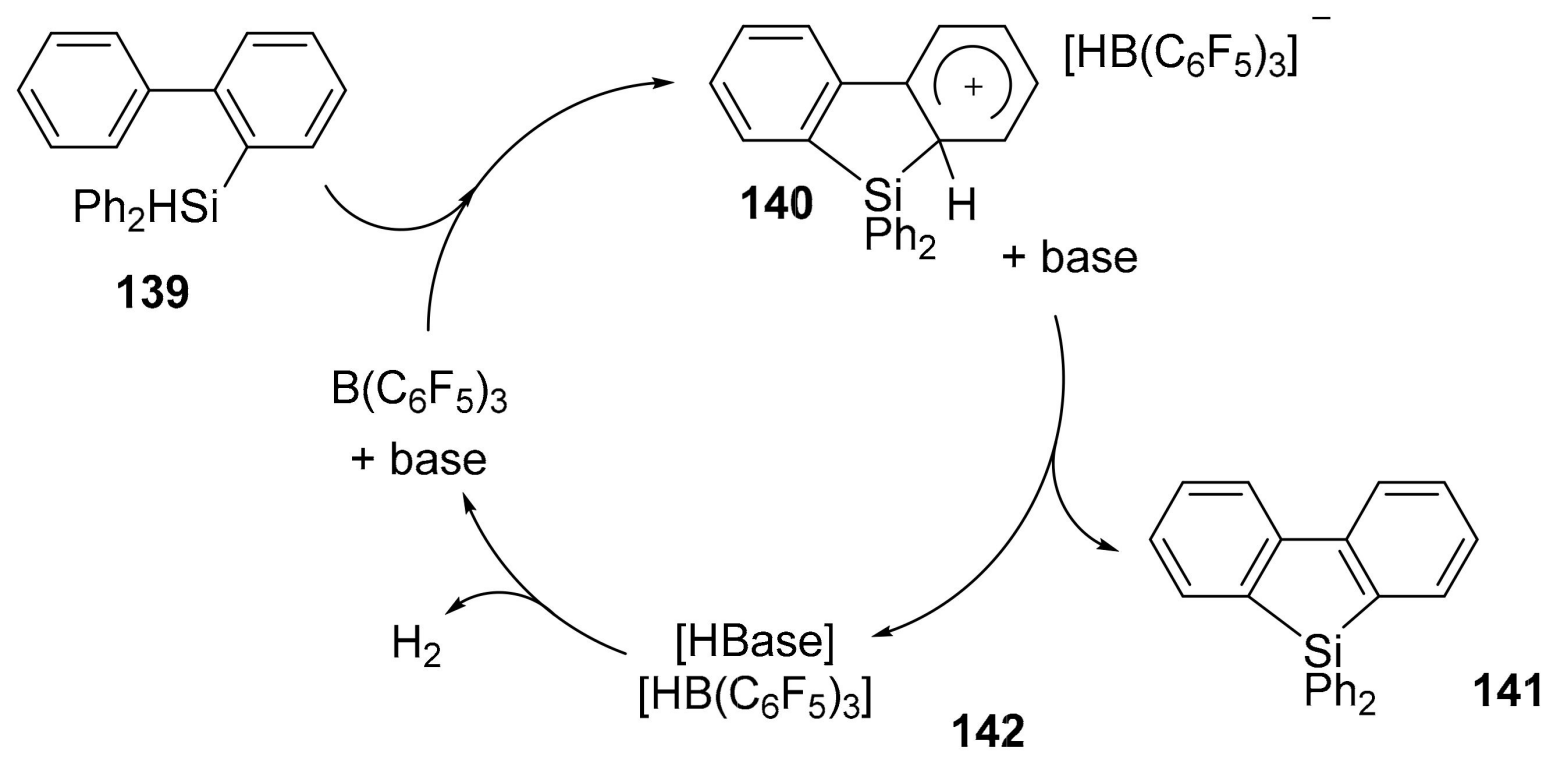
Possible mechanism for the synthesis of dibenzosilole derivatives via a sila-Friedel-Crafts reaction.

The main limitations of this sila-Friedel-Crafts reaction are the requirement of a stoichiometric amount of Ph_3_CB(C_6_F_5_)_4_ reagent and 2,6-lutidine base. Moreover, the yields of these reactions are low and need to be improved. Notably, Curless and Ingleson reported an efficient method using B(C_6_F_5_)_3_ as catalyst to activate silanes **139** and 2,6-dimethylpyridine as base to facilitate deprotonation of the silylated arenium cation **140**. A plausible catalytic cycle for the formation of 9-silafluorene **141** was proposed (Scheme 33). In addition, this method has also been successfully applied in the synthesis of benzosilole derivatives from the corresponding alkynes via a Ph_3_CB(C_6_F_5_)_4_ catalyzed one-pot hydrosilylation/dehydrosilylation process [59].

#### 2.5.3. Cyclizations via Silyl Radicals

**Scheme 34:**
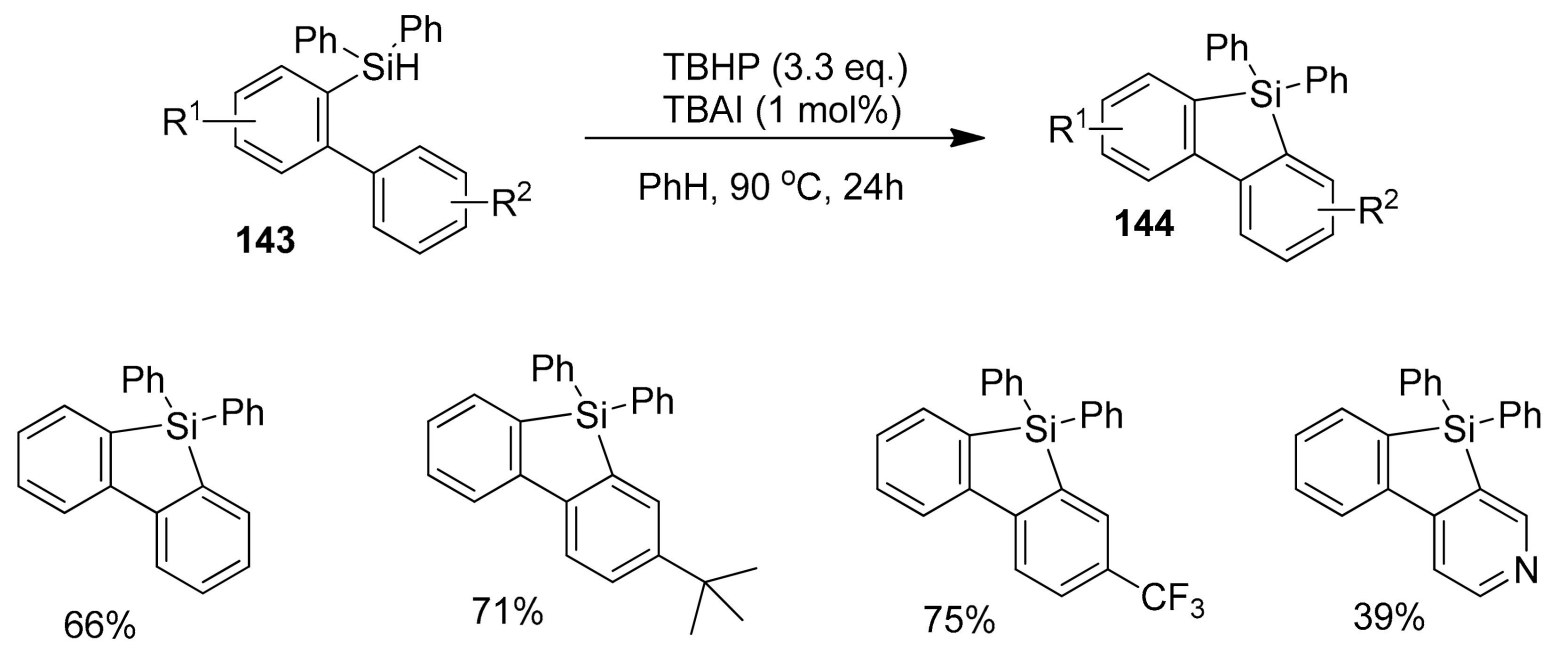
Synthesis of dibenzosilole derivatives via silyl radicals.

**Scheme 35:**
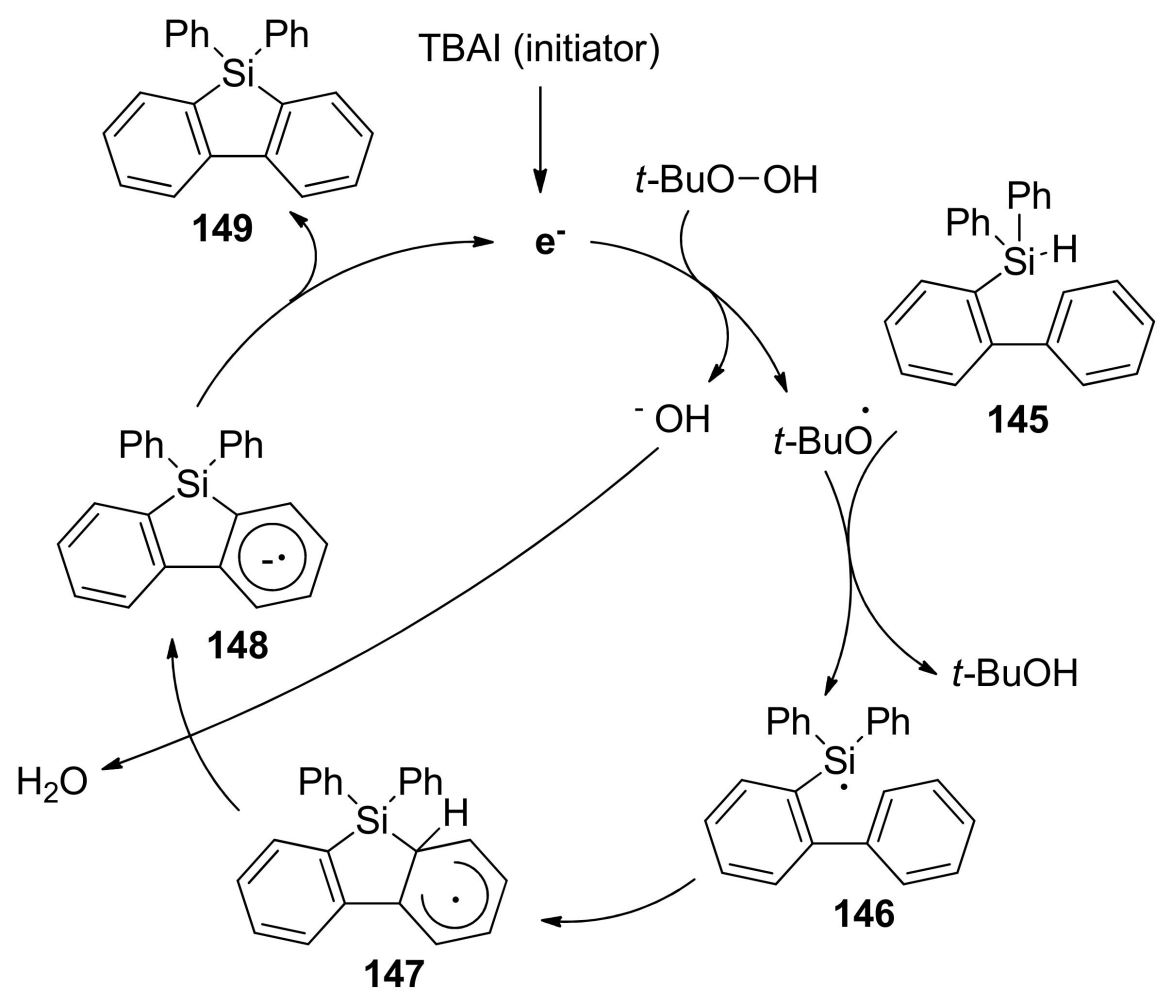
Proposed mechanism for the synthesis of dibenzosilole derivatives via silyl radicals.

Recently, several “green” approaches have been reported to prepare benzosilole derivatives without using expensive and toxic transitions metal catalysts [60]. In 2015, Leifert and Studer disclosed a transition metal-free synthesis of 9-silafluorenes **144** by intramolecular radical silylation of 2-diphenylsilylbiaryls **143** via base-promoted aromatic substitution (Scheme 34) [60]. The use of *tert*-butylhydroperoxide (TBHP) as a cheap stoichiometric oxidant in the presence of a small amount of tetrabutylammonium iodide (TBAI) is necessary for the success of this reaction. The cyclization reaction via a silyl radical process was performed under mild conditions, affording good yields of the desired products **144**. A plausible mechanism for the synthesis of 9-silafluorene **149** was proposed, in which a silyl radical **146** was generated by reaction of the substrate **145** with *tert*-butoxyl radical as the key intermediate (Scheme 35). In this process, TBAI may act as an initiator and not as a catalyst.

**Scheme 36:**
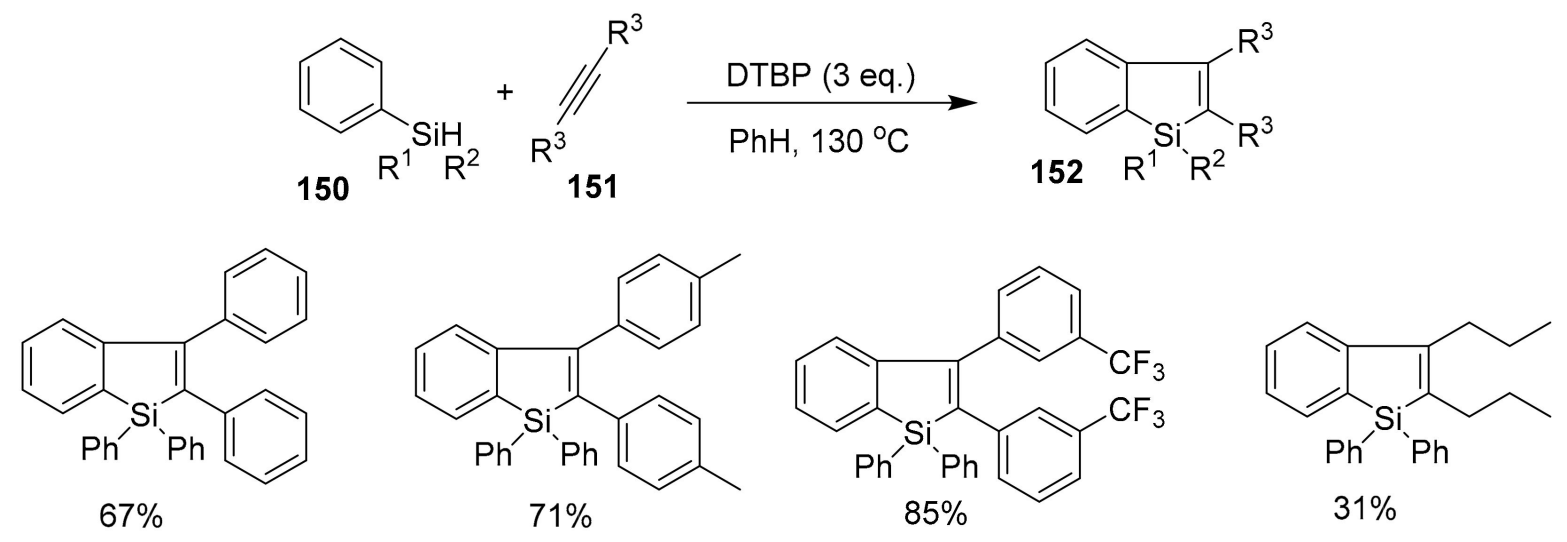
Synthesis of benzosilole derivatives via silyl radicals.

In 2015, Li et al. reported a similar metal-free synthesis of benzosiloles **152** and 9-silafluorenes **144** in good yields from arylhydrosilanes **150** and alkynes **151** via a silyl radical process using DTBP as the radical initiator (Scheme 36) [61]. PhCF_3_ was found to be the most suitable solvent for the success of these transformations. These catalyst-free processes may open new sustainable approaches to prepare benzosiloles, 9-silafluorenes and π-extended benzosilole derivatives in near future.

**Scheme 37:**
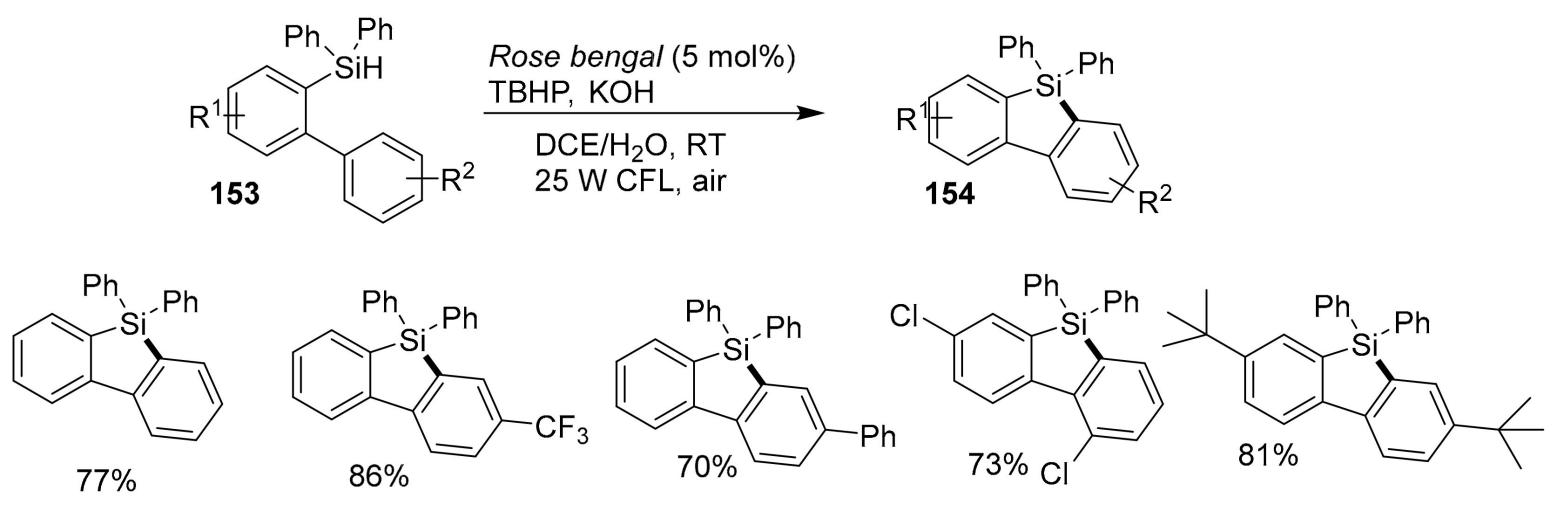
Synthesis of dibenzosiloles from biarylhydrosilanes via a visible-light induced radical silylation.

**Scheme 38:**
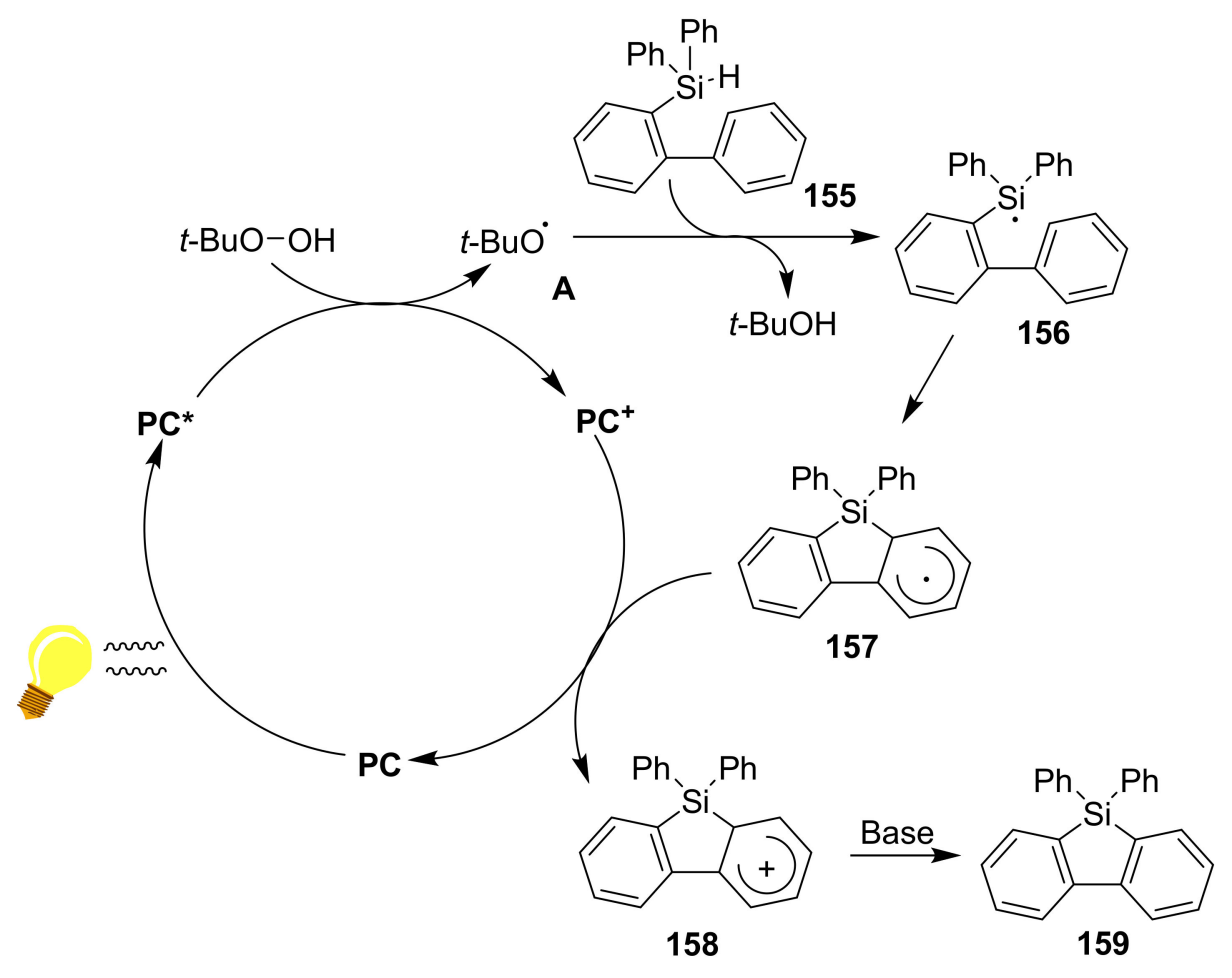
Proposed mechanism for the synthesis of dibenzosiloles from biarylhydrosilanes via a visible-light induced radical silylation.

Recently, visible-light photoredox catalysis has become one of the most important tools for developing new organic transformations due to its advantages such as the high tolerance to functional groups and mild operating conditions [62,63]. In 2017, Jiang et al. disclosed an efficient synthesis of dibenzosiloles **154** from biarylhydrosilanes **153** via a visible-light induced radical silylation process. In the presence of rose bengal as photocatalyst, dibenzosilole derivatives were prepared in good yields at room temperature under water/air compatible conditions (Scheme 37) [64]. Several studies on light/dark experiments and quantum yield measurements provided valuable evidences supporting the proposed photocatalytic mechanism from **155** to **159** (Scheme 38).

## 3. Conclusions

In this paper, synthetic methods and important physical properties of dibenzosiloles, heterocycle-fused benzosiloles and π-extended benzosiloles have been systematically reviewed. Most of significant research in this field relies on the development of transition metal-catalyzed processes which were developed over the last decade. Several reports on transition metal-free catalytic processes also mentioned some promising results. Especially, the visible-light induced radical silylation process using catalytic amount of rose bengal sensitizer gave 9-silafluorenes in good yield at room temperature under mild condition. Up to now, many novel π-extended benzosiloles have been designed, synthesized and characterized. The fast growth of this field has opened new pathways in the development of novel advanced organic materials such as OLEDs, photovoltaic devices, and semiconductors with outstanding electronic and photophysical properties. In addition, benzosiloles, dibenzosiloles and π-extended fused benzosiloles have been successfully used as key building blocks in the design of many important polymer/copolymer materials.
